# Analysis of the development of children discharged from the Neonatal Intensive Care Unit from parents’ point of view

**DOI:** 10.1590/0034-7167-2022-0717

**Published:** 2023-11-27

**Authors:** Lediana Dalla Costa, Késsia Helen Dalmuth, Mayeli Thais Fernandes Vieira, Emanueli Girardi, Gisely Fachinello, Ângela Morais da Silva, Jolana Cristina Cavalheiri, Alessandro Rodrigues Perondi

**Affiliations:** I Universidade Paranaense. Francisco Beltrão, Paraná, Brazil; II Hospital Regional do Sudoeste Walter Alberto Pecóits. Francisco Beltrão, Paraná, Brazil

**Keywords:** Neonatal Nursing, Child Development, Infant Mortality, Intensive Care, Neonatal, Newborn, Enfermería Neonatal, Desarrollo Infantil, Mortalidad Infantil, Cuidado Intensivo Neonatal, Recién Nacido, Enfermagem Neonatal, Desenvolvimento Infantil, Mortalidade Neonatal, Terapia Intensiva Neonatal, Recém-Nascido.

## Abstract

**Objectives:**

to analyze the development of children discharged from the Neonatal Intensive Care Unit and how hospitalization interferes with child development, from parents’ point of view.

**Methods:**

a descriptive-exploratory, documentary and qualitative study, carried out with parents of children discharged from the Intensive Care Unit through a telephone survey and supplementation in medical records. Data were analyzed using Bardin’s tool and discussed according to scientific evidence.

**Results:**

from parents’ point of view, delay in physical development was not observed, however difficulties in breastfeeding and weight gain were reported. Changes in speech and changes in behavior considered abnormal were also delays in child evolution.

**Final Considerations:**

from parents’ point of view, changes in speech may be associated with hospitalization in an Intensive Care Unit, while difficulties related to breastfeeding and delays in cognition may be altered by conditions of prematurity and low weight at birth.

## RESUMO

**Objetivos:**

analisar o desenvolvimento de crianças egressas da Unidade de Terapia Intensiva Neonatal e como a internação interfere no desenvolvimento infantil, na visão dos pais.

**Métodos:**

estudo descritivo-exploratório, documental e qualitativo, realizado com pais de crianças egressas do setor intensivo por meio de inquérito telefônico e complementação em prontuário. Os dados foram analisados pela ferramenta de Bardin e discutidos conforme as evidências científicas.

**Resultados:**

na visão dos pais, não se observou atraso no desenvolvimento físico, entretanto dificuldades no aleitamento materno e no ganho de peso foram relatados. Alterações na fala e mudanças no comportamento consideradas anormais também foram atrasos na evolução da criança.

**Considerações Finais:**

na visão dos pais, as alterações na fala podem ser associadas à internação em unidade intensiva, enquanto dificuldades relacionadas ao aleitamento materno e atrasos na cognição podem se mostrar alteradas pelas condições de prematuridade e baixo peso no momento do nascimento.

## RESUMEN

**Objetivos:**

analizar el desarrollo de los niños egresados de la Unidad de Cuidados Intensivos Neonatales y cómo la hospitalización interfiere en el desarrollo del niño, en la visión de los padres.

**Métodos:**

estudio descriptivo-exploratorio, documental y cualitativo, realizado con padres de niños egresados de la Unidad de Cuidados Intensivos a través de encuesta telefónica y complementación en prontuarios. Los datos fueron analizados utilizando la herramienta de Bardin y discutidos de acuerdo con la evidencia científica.

**Resultados:**

a juicio de los padres, no se observó retraso en el desarrollo físico, sin embargo, se relataron dificultades en la lactancia y aumento de peso. Los cambios en el habla y cambios en el comportamiento considerados anormales también fueron retrasos en la evolución del niño.

**Consideraciones Finales:**

a juicio de los padres, las alteraciones del habla pueden estar asociadas a la hospitalización en una Unidad de Cuidados Intensivos, mientras que las dificultades relacionadas con la lactancia y los retrasos en la cognición pueden verse alterados por condiciones de prematuridad y bajo peso al nacer.

## INTRODUCTION

Intensive Care Units (ICU) are one of the essential components of modern medicine that currently exists. They stand out for being complex hospital units, with continuous care and advanced technology, providing varied and intensive care, depending on the severity of the disease and/or the condition of admitted patients^(1)^.

Among the subtypes of ICU, the Neonatal Intensive Care Unit (NICU) is mentioned, an environment specially designed for newborns (NB) who need assistance and continuous complexity, such as preterm and low birth weight infants^(2)^. These units provide comprehensive services to NB, a structured environment with the provision of specialized services for the different conditions of admitted patients. There are multidisciplinary teams and great technology available for safe and effective care^(3)^. Hospitalizations in these units are usually long, favoring greater exposure of NB to risks and intercurrences, due to excessive manipulations, multiple monitoring equipment and performed procedures they receive^(2)^.

According to the Manual of Neonatology of the State Department of Health^(4)^, the neonatal period is from birth to 27 days, 23 hours and 59 minutes, and the early neonatal period is from birth to six days, 23 hours and 59 minutes, and the late neonatal period from the seventh day until reaching 27 days, 23 hours and 59 minutes. To classify NBs’ gestational age, the manual defines as preterm those born at less than 37 weeks of gestation; term, those born between 37 and 42 weeks; and post-term, those born at more than 42 weeks of gestation.

There are many factors reported in the literature on NB hospitalizations in the NICU. A study carried out in the Brazilian Health System (*Sistema Único de Saúde*) in São Paulo showed that hospitalization occurs due to factors considered high risk, such as: mothers aged >35 years; inadequate prenatal care; obstetric complications; cesarean delivery; prematurity; low weight at birth; Apgar <7 and congenital malformation. There is a prevalence of hospitalizations of premature NB^(5)^, which may have impaired child development or neonatal death^(6)^. There are several body systems of preterm NB that are immature at birth, such as the respiratory, digestive and body temperature systems, in which monitoring and intensive care are necessary^(7)^.

Human development is divided into three main domains, physical, cognitive and psychosocial, which are assessed according to the age groups of the development cycle, which describe the changes seen in each phase. These cycles of age groups are divided into eight, tracing the profile visualized in each of them: the prenatal period, early childhood, second childhood, third childhood, adolescence, adult life, intermediate adult life and third age^(8)^.

A study carried out with adults shows that, after their hospitalization in the ICU, impairment of physical function and partial or complete dependence for activities were observed, and that these consequences can continue for five years or more after leaving the ICU^(9)^. However, with NB, one carried out in Botucatu-SP also showed that prematurity was a risk factor for hospitalization in the NICU, but that this factor is not associated with the use of other health services during the first 12 months^(10)^. In this last research, follow-up and assessment were limited to the first year of life after discharge, not allowing to have a vision of how children can be after these first 12 months studied.

When considering the few researches in the area and the significant number of hospitalizations in NICUs in Brazil, this study has the following questions: how is the development of children discharged from the NICU? Does NICU admission interfere with child development from parents’ point of view?

## OBJECTIVES

To analyze the development of children discharged from the NICU and how hospitalization interferes with child development, from parents’ point of view.

## METHODS

### Ethical aspects

The research was approved by the Research Ethics Committee of the *Universidade Paranaense*, and complies with the ethical principles set out in Resolutions 466/12 and 520/16 of the Brazilian National Health Council. The approval of the institution where the data from the medical records was collected and Informed Consent Form (ICF) acceptance, available online on Google Forms, were also considered.

### Study design

This is a descriptive-exploratory, documentary and qualitative study, carried out through a telephone survey with parents and guardians of children discharged from the NICU and data collected from medical records. For its development, the COnsolidated criteria for REporting Qualitative research (COREQ) checklist criteria were followed^(11)^.

### Methodological procedures

The telephone survey applied followed a collection instrument in the form of a questionnaire, consisting of variables about the children and the respective guardian and guiding questions related to child development during childhood and post-hospitalization. Data collection from medical records was complementary to the interview, taking into account the variables related to childbirth and the period of children in the NICU.

For sample selection, children who left the NICU in the period 2011-2021 were used, and participant selection was carried out through a list made available by the institution’s psychology service, which has the telephone contact of parents and guardians to exchange information about children’s conditions during hospitalization.

With the intermediation of these professionals, guardians were invited to participate in the research, providing the researchers’ telephone contact to parents so that those who were interested in participating in the study could express their acceptance and organize data collection. The interview took place through telephone contact made by the researchers, with audio recording. Each interview lasted an average of 30 minutes.

### Study setting

The study site was a public hospital located in the municipality of Francisco Beltrão-PR, Brazil, which covers 42 municipalities in southwestern Paraná, being a reference in polytrauma and high-risk pregnancy, serving more than 2,000 patients per month and which has a NICU about ten years ago.

The research site was chosen because it is the only neonatal intensive service in southwestern Paraná, having 10 intensive beds and 5 intermediate care beds with a multidisciplinary team and technological structure to support NB’ needs, including receiving patients from different regions of the state. Care is carried out according to children’s needs, through the regulation and availability of beds in the institution, regardless of the risk stratification assigned to pregnant women during prenatal care.

### Data source

It started with a literature review in order to identify the profile of NICUs in Brazil. Afterwards, the researchers developed a data collection instrument divided into two parts, the first being applied during the interview with parents and guardians who agreed to participate in the research, and the second containing data regarding ICU stay.

The study had the participation of 15 subjects, with two dropouts, and the sample consisted of 13 guardians. Regarding the number of children investigated, there was one case of twins and one of triplets, resulting in 16 children surveyed.

Parents and guardians whose children were born in the period 2011-2021, admitted to the NICU of the researched institution and who were discharged from the hospital, were included. Parents and guardians whose children died before discharge from the service were excluded from the study. Also, parents and guardians who requested to withdraw from the study were discarded.

### Data collection and organization

Data collection took place from June to August 2022, with prior scheduling of the interview with guardians, according to preference. As an instrument for collecting data from the interviews, a script prepared by the researchers was used, which included variables related to children and parents, such as age, height, weight, existing comorbidities and medications being used by the child, employment of guardians, approximate per capita family income, child gender, multidisciplinary and childcare follow-up, and childhood vaccination. Thus, it was guided by open-ended questions: after discharge from the NICU, how was the child’s growth and development? What interferences were observed in your child’s development due to use of NICU? How do you rate NICU care and what guidelines would you give to parents whose children need to be admitted to this unit?

Afterwards, data were collected from medical records with variables related to childbirth and children’s stay in the ICU, such as maternal age, gestational age, birth weight, Apgar score, type of delivery, reason for hospitalization in the NICU, length of stay and hospital outcome.

After collection, the interviews were transcribed in their entirety for data analysis. Study participants were identified as G01, G02, G03 and so on, with R referring to the initial letter of the word “Guardian”, and the following number to the order in which the interviews were carried out.

### Data analysis

The content analysis process was carried out using Bardin’s tool, which is divided into three phases: pre-analysis, material exploration, treatment of results. Data analysis occurred simultaneously with data collection. Each interview was examined individually, sentence by sentence, to separate the data into distinct parts and compare them, in search of similarities and differences, identifying and separating by categories. The categories obtained were guided by the division of domains of human development^(8)^.

## RESULTS

Of the children studied, there was a prevalence of males (75%), belonging to early childhood (82.3%). The main reasons for admission to the NICU were prematurity (93.8%) and low birth weight (87.5%). Of the parents interviewed, 62.5% reported that their child did not have chronic diseases. All underwent multidisciplinary follow-up after admission to intensive care, with emphasis on high-risk pediatrics (81.3%) and physiotherapy (43.8%).

Based on the interviews carried out, child development was observed in the three stages of childhood, thus allowing four categories to be delineated: *Physical development; Cognitive development; Psychosocial development*; and *Feelings experienced by parents*. The first three were guided by the domains of human development^(8)^.

### Physical development

Regarding child physical development, most parents reported not perceiving delays, but development according to child’s age, regardless of the complications suffered during the period in the NICU or the reason for which they were admitted. The reports also included the multidisciplinary follow-up after discharge, essential to monitor child evolution, and the possible sequelae that the child could develop due to the initial diagnosis.

[...] *the question of development such as crawling, sitting, walking, cervical control, trunk control, in short, everything was within the corrected age. It was all right.* (G06)

[...] *the day he turned eight months old, he really started to crawl, he started to walk when he was a little over a year old, a year and twenty days old or so, and then, after that, he always, it was all the signs, the signs of readiness* [...]. (G09)

Some guardians reported that other health professionals talked about possible atypical development, preparing parents for possible post-discharge complications, but these changes were not observed by parents in the three childhood stages of the children studied.

[...] *walk, he walked at the right time, the doctor explained that everything would be fine later, for him to talk, for him to walk, crawl, but we went there, the doctor had that surprise* [...]. (G12)

[...] *even the orthopedist said that he probably wouldn’t walk or it would take him a long time to walk, but thank God he did physical therapy, he walked at one year and seven months.* (G13)

There were few problems reported by those responsible for children’s physical development, and one of the most observed was breastfeeding in the first months they were at home. This difficulty was associated by parents with prolonged hospitalization time and the feeding inside the unit, since the milk was offered by tube or by the cup. Unfortunately, although the institution is recognized regionally and is the only NICU, it does not have a milk bank, but the multidisciplinary team provides all the support and encouragement for mothers to express milk and make breast milk available to children. As a result of the difficulty in introducing breastfeeding, weight gain impairment was evident in the first years of life, favoring the early introduction of other types of milk, food and infant formulas.

[...] *Another point that we are running a lot behind is the weight issue, no, he does not gain the correct weight that he needs to be at two years old, he just weighs ten kilos* [...]. (G06)

[...] *she gains weight not so fast, like a child who is born at nine months old, it is slower, but she, like, is very smart.* (G08)

Despite the small difficulties encountered, the interviewed guardians reported that they had or are following up with the multidisciplinary team both in the city where they live and in the outpatient clinic and in the regional center of specialties, being referred by the egress hospital institution and receiving guidance on the post-discharge period. Also, they reported that stimulation and monitoring influence the good development that children present.

### Cognitive development

It was observed that some parents mentioned cognitive adaptation without irregularities, such as memory, learning, expressions and communication, again reporting the influence of the multidisciplinary follow-up they had after discharge. This is a positive factor, as it demonstrates that hospitalization in the NICU does not compromise children’s cognitive development in the studied sample and that monitoring the post-discharge evolution can be great in the early detection of intercurrences.

The greatest difficulty observed and reported was speech development, in which most parents observed a delay, even when compared to older siblings or children close to the family.

[...] *talk, as I have another one who is older, she* [the child], *like, it took a little longer than the other one, but she spoke before two years old yet.* (G11)

[...] *only in speech, like, she is taking a little longer, she takes longer to speak, it is taking longer to speak, but she says “mommy”, “daddy”, “baby”, the “au-au”, there is a dog called Toddy, she speaks Toddy very well. So, like, we help her a lot too, but, like, in the speech, I found it a bit time-consuming.* (G08)

[...] *now he is only two years old and has a delay in speech, but he is already being followed up with a speech therapist, he is also being followed up with a physio* [...]. (G06)

### Psychological development

Of the guardians interviewed, most recognized that children had already assumed his own personality. Among the addendums made by the parents, some reported that the child had episodes of irritation above what they considered normal, but that, despite this, they easily adapted to the environment in which they were inserted.

Not all of them are attending daycare/school, because, due to the COVID-19 pandemic, some parents chose to stay with their child at home or under the care of a third person, however, in relation to those who attend school, parents show that there are no complaints, having many positive feedbacks from teachers and collaborators. The relationship with other children and adults was also no different, and the interviewees highlighted excellent adaptation. Only two observed difficulties in psychosocial development, with one responsible reporting signs of obsessive-compulsive disorder (OCD), and another, the investigation of an attention deficit disorder with hyperactivity (ADHD) with professional follow-up.

[...] *and he is very, with a lot of personality, a lot, a lot of personality, he is very, I think, I always worry, because he is a two-year-old baby, but, and he has children that he has contact with every day, and now, at daycare and everything, and even here at home, I am surrounded by children around the house, but he is a very, very adult.* (G10)

[...] *he is very angry, even to exchange him I take him to a dance.* (G01)

[...] *it’s for writing, he has, he doesn’t have beautiful handwriting like most children, he has spatial difficulties, yeah, and we investigate an ADHD* [...]. (G13)

### Feelings experienced by parents

During the interviews, several parents expressed the feelings they experienced or still experience with the NICU experience. Some parents were moved when talking about the struggle they faced from birth to adaptation with children in the post-discharge process.

A mix of feelings was reported during the interviews, such as unpreparedness to be “ICU parents”, something often seen in the neonatal context, and a feeling of uncertainty about child evolution, due to the experience heard from third parties.

[...] *in fact, I think it was more fear than that, because, as he was very small, we were very afraid that he would not survive.* (G13)

[...] *for me, it was good, I was very calm knowing that she was going to the ICU, at that moment, I knew that she would be fine.* (G04)

Fear and stress were the most expressed feelings, due to adapting to that new phase inside the hospital, with the uncertainty of prognosis along with birth fragility and the complications that may happen outside hospital environments, in which children would not have assistance from the ICU, in case something happened. These feelings lasted during child development and influenced parents in child care and monitoring. On the other hand, team reception and the complexity of the sector comforted and gave hope for a positive prognosis of hospitalized children.

## DISCUSSION

In this research, the objective was to analyze the child development of children discharged from the NICU, according to the perception of parents and guardians. This study covered children of the first, second and third childhood, prevailing those of the first. The main causes of hospitalization in the unit were prematurity and low birth weight, two risk factors that prevail in NB hospitalization in the NICU^(5,12)^.

According to the perception of those in charge of the physical development after discharge from the NICU, assessment was positive, denying intercurrences and reporting good development, according to child’s age. The result found differs from the study carried out in North American NICUs, in which four times greater risks of motor delay were associated in children who underwent intensive care in the first two years of life^(13)^; however, it corroborates the Brazilian study carried out in the outpatient clinic of a public hospital in Curitiba-PR, from 2017 to 2018, where it was observed that, of the children monitored, 79% presented normal development in the first year, and those who presented deficits in the first moment they overcame the delays with time. In this same study, the multidisciplinary approach and the stimulus provided by parents stand out as essential in post-discharge development^(14)^.

A positive child development is influenced by several variables in which the NB or the child experiences, and this includes the environment in which they are inserted, the food offered and among other conditions. Exclusive breastfeeding is recommended until the sixth month of life, as it has essential nutrients for baby development^(15)^. In this context, a study carried out in Rio Grande do Sul sought to know the prevalence of breastfeeding offered within the intensive care environment and found a low number of children fed with exclusive breast milk, which may be related to hospitalized children’s low weight^(16)^.

Breast milk has proteins and minerals that contribute to children’s immune formation, which is a vulnerable system in children who are hospitalized in intensive care, becoming a risk factor for the development of diseases and infections^(17)^. Moreover, breastfeeding is fundamental in premature and underweight child development, as it contributes to the increase of antibodies, weight gain, reduction of respiratory diseases, malnutrition, morbidity and mortality and neuromotor and cognitive function improvement^(18-19)^.

Children’s cognition had few intercurrences observed by mothers, and development according to age was also reported and that, in the school phase, adaptation and progress receive positive reviews by teachers. This result contradicts a study carried out in 2004, in the city of Rio de Janeiro, with NICU graduates who were already in the preschool phase, in which an intelligence quotient (IQ) was identified with an average below the normal range and a high incidence of children with cognitive impairment with impaired functioning in planning, thinking, perceiving, orientation, and memory functions; this same study considered the use of intensive therapy in the studied children’s atypical development^(20)^.

However, with regard to the use of intensive care, another study carried out with 109 children aged up to 10 years, in the city of Rio Grande-RS, Brazil, showed that the length of stay in the ICU did not influence the performance of children who took the test and that this is not a risk factor for learning difficulties, breaking the idea that the ICU and graduates have lower academic performance^(21)^. This result is in line with this study and, when comparing the difference in years between one study and another, it can be considered that there have been technological advances and studies in the area, with improvement in neonatal interventions and professional qualification, optimization provided by the NICU to graduates, which has guaranteed a better quality of life and almost zero influence on school development.

Speech delays were perceived by most mothers, reporting that children began to pronounce their first words around two years of age or even later, even with those who were followed up by the specialized team. Linguistic speech is influenced by perinatal factors to which the child is exposed, such as low weight, prematurity, neonatal asphyxia and breastfeeding, and, as a result of these, a delay in the onset of syllable formation and manifestations can be observed, depending on the age group^(22)^. It is estimated that the first formed syllables begin around ten months and, at the end of the first year, the pronunciation of the first words, reaching 18 months with an approximate vocabulary of 50 words^(8)^.

Perceived delay may be associated with intensive care and invasive procedures performed, since, in addition to the immaturity of body systems, it may result in impairment of neurodevelopment, such as vision, hearing, speech and communication. These results are related to impaired development of brain systems that are often affected by insults during the perinatal period, such as premature birth and its complications to adverse events experienced in the NICU, such as metabolic and respiratory changes, compromising learning throughout childhood^(23)^.

A study carried out in New Haven and Providence, in the period 1998-1999, with 440 children, indicated that prematurity is associated with psychosocial deficits, showing children who are more withdrawn, inattentive, aggressive and with disturbances in the thought process^(24)^, corroborating the results found in this study, in which characteristic personifications perceived by mothers were reported, in addition to accentuated emotions, such as extreme irritability.

The investigation of ADHD and OCD was also mentioned, reporting atypical psychosocial development. In addition to this, behaviors characteristic of neurobiological disorders, such as anxiety and hyperactivity, were cited. This finding is in line with a study carried out in 2012, in the interior of São Paulo, with 29 children enrolled in the early childhood education network, which interviewed parents and teachers about the psychosocial development of those surveyed, and observed changes in children’s behavior at home and inside the school. According to this study, hyperactivity and relationship problems with friends were the highlights mentioned by parents, followed by emotional and conduct problems; by teachers, on the other hand, hyperactivity and emotional changes were the most observed in the classroom^(25)^. In common, all children were born prematurely and with low weight, establishing an association of these factors with potential signs of ADHD and developmental coordination disorder (DCD)^(25)^.

Along with uncertainties about child development and impasses experienced during the hospitalization period and even after it, the feeling that parents experience in this phase marks them beyond the moment of discharge. Reports of anguish, fear and suffering gave way to hope and confidence, as they saw their children evolving during hospitalization. Knowing that their children were in a higher quality care facility comforted and supported the parents, who also reported relief from the attention and care provided by the multidisciplinary team^(26-27)^.

This scenario reinforces the importance of support from the multidisciplinary team to parents who are often vulnerable and unprepared to face this moment, needing emotional support, clarification and confidence about hospitalization and evolution^(28-29)^. In an interview with parents in Rio Grande do Norte, they reinforced the importance of the support provided by the health team, highlighting the role of nursing, which provides humanization and facilitates the experience in intensive care environments^(30)^.

Another interview carried out in the city of Brasília - Federal District showed feelings of hope and positivism regarding the ICU stay, highlighting the wait for a positive recovery and survival in the face of technological resources, the complexity of the environment and the expertise of the professionals who work in this sector, highlighting this positive feeling and once again associating the trust passed on by the professionals who work in this environment^(31)^.

### Study limitations

This study has as limitations its sample size, consisting of 13 parents interviewed. In addition, although the researched institution is a reference in neonatal care in the region, the results cannot be generalized, representing only the outcome of a group of participants, limiting knowledge of other realities in this context.

### Contributions to nursing

The results obtained through this research contribute to nursing in a relevant way, as they allow viewing the development of children discharged from intensive care and the areas of child development that may have been affected by hospitalization. It is hoped that this study will contribute to increasing the visibility of this theme and of researches that glimpse the look of parents in their adaptation at home with their children as well as help them to detect early changes in development, which can contribute to minimize doubts, fears and uncertainties reported by parents in this study.

## FINAL CONSIDERATIONS

From the analysis carried out, it was possible to observe that the investigated children were in different stages of growth and child development, and there were few alterations listed by the mothers during child evolution. It was observed that prematurity and low weight were the main causes for NB admission to ICU, which, after hospital discharge, maintained multidisciplinary follow-up.

As for physical development, the parents did not perceive delays in the children in reaching the assessment milestones for each age, and, after discharge, they experienced difficulties in breastfeeding and consequent weight gain. In cognitive development, delays in language development were reported, while, in psychosocial, there were changes in behavior considered abnormal by parents, such as extreme irritability and potential for the development of learning and mental disorders.

Furthermore, they also reported feelings of fear, insecurity and anguish in the face of the conditions experienced, but which were mitigated by knowing that children were amidst the technological and complex advancement that the NICU provides. Welcoming parents by the team during hospitalization proved to be favorable and important for their experience in the NICU, instilling confidence in a process of improvement for children.

From parents’ point of view, speech disorders may be associated with hospitalization in an ICU, while physical delays related to breastfeeding and delays in cognition may be altered by conditions of prematurity and low weight at birth. It is reiterated that this positive factor may be associated with the quality care provided within the unit, the advanced technology as well as the continuous monitoring and encouragement by the country, proving to be effective in adequate child development.

## Supplementary Material

0034-7167-reben-76-05-e20220717-sup01

## Data Availability

https://doi.org/10.48331/scielodata.9QDQFR
