# Supplementary material for: Analysis of the development of children discharged from the Neonatal Intensive Care Unit from parents’ point of view
Source: Rev Bras Enferm. 2023 Nov 27;76(5):e20220717. doi: 10.1590/0034-7167-2022-0717 (PMC10680386; doi:10.1590/0034-7167-2022-0717)
Supplement: 0034-7167-reben-76-05-e20220717-sup01 [file 0034-7167-reben-76-05-e20220717-sup01.pdf]

INSTRUMENTOS DE COLETA DE DADOS

<table>
<tr><td colspan="3">PRONTUÁRIO DA MATERNIDADE:
RN</td></tr>
<tr><td>Idade da mãe</td><td colspan="2"></td></tr>
<tr><td>Idade gestacional ao nascer</td><td colspan="2"></td></tr>
<tr><td>Apgar no 1º e 5º minuto de vida</td><td>1º</td><td>5º</td></tr>
<tr><td>Peso ao nascer</td><td colspan="2"></td></tr>
<tr><td>Tipo de Parto</td><td>Natural ( )</td><td>Cesária ( )</td></tr>
<tr><td>Motivo do Internamento em UTIN</td><td colspan="2">( ) Prematuro ( ) baixo peso ( ) SDR ( ) Icterícia
( ) Cardiopatia ( ) Infecção ( ) Apgar <7 ( ) GIG
( ) D. Renal ( ) Tocotraumatismo ( ) Mal formação
( ) PCR ( ) Hipoxia ( ) Hepatite Perinatal
( ) Gastroquise ( ) outro ______________________</td></tr>
<tr><td>Terapêutica Instituída</td><td colspan="2"></td></tr>
<tr><td>Tempo de Permanência UTIN</td><td>Dias</td><td>meses</td></tr>
<tr><td>Prognóstico (Doenças crônicas, medicamentos, acompanhamentos)</td><td colspan="2">( ) Alta ( ) Acompanhamento</td></tr>
<tr><td colspan="3">Considerações Finais
____
____
____
____
____
____
____

DATA: ____/_____________/____</td></tr>
</table>

**ENTREVISTA COM OS PAIS/ CRIANÇA**<br>**RN ___________**		
Idade Atual Criança		
Altura:		
Peso:		
Doenças:	( ) Não<br>( ) Sim, quais________________________________	
Medicamentos em uso		
Trabalho Materno e Paterno	( ) Autônomo ( ) Estágio ( ) Doméstico ( ) Voluntário ( ) Eventual ( ) Avulso ( ) Temporário ( ) Desemprego	
Renda *per capita* familiar	( ) >=1 salário mínimo ( ) 2-3 ( ) 4-5 ( ) + 5 salários	
Gênero da criança	( ) F	( ) M
Acompanhamento puericultura	( ) Sim	( ) Não
Vacinação	( ) Sim	( ) Não
1 - Após a alta na UTIN, como foi o crescimento e desenvolvimento da criança?		
2 - Quais interferências foram observadas no desenvolvimento do seu filho devido a utilização da UTI Neonatal?		
3 - Como você avalia o atendimento da UTI Neonatal e quais as orientações você daria aos pais cujos filhos necessitam de internamento nessa unidade?		

ENTREVISTAS TRANSCRITAS DE APARELHO MP3

Para manter a privacidade, os participantes foram identificados aqui como:
R: Responsável
E: Entrevistadora

R01:

E: Ele tem que idade?

R01: Um ano e sete meses

E:Sabe me dizer quanto ele está medindo e pesando?

R01: Pesando sim 9,60kg, mas não lembro da altura

E: Ele tem alguma doença crônica mãe? ou faz uso de algum medicamento de uso contínuo?

R01: Ele teve uma cirurgia para retirar um rim que tinha mal formação, e têm broquiolite, usa bombinha Aerolin

E: Faz tempo q ele fez a cirurgia?

R01: Assim ele nasceu com 31 semana, e com trinta dias por aí já fez

E: Foi feito acompanhamento de puericultura com ele no postinho de saúde? A vacinação também está em dia?

R01: A vacinação tá em dia, mas puericultura não. Tive muita correria indo para Curitiba, até quatro vez por mês, o trabalho e a pandemia, daí não estavam fazendo aqui na nossa cidade

E: A sua renda é de quantos salários?

R01: 1.230

E: Após a alta da UTI Neonatal, como tem sido o crescimento e desenvolvimento do seu filho?

R01: Sabe, desenvolveu bem assim, crescimento tudo, só tem um atraso com ele assim, que ele ainda não está caminhando sozinho ainda, mas no mais tá tudo perfeito Gracas a Deus.

R01: Claro né, quando a gente vai pra Curitiba ele tem feito vários exames, pra ver como que está tudo, tá tudo certo graças a Deus. Agora, em agosto ele tem que fazer uma RM, mas está tudo certo até agora, todos os exames que fez, tudo certo.

E: faz acompanhamento com pediatra ou com outros profissionais tbm (fisioterapia, psicologo)?

R01: Sim com quatro profissional lá em Curitiba, aqui com pediatra.

E: Ele compreende quando falam com ele, quais as reações dele?

R01: Sim, ele tá começando a falar algumas coisas, e entende tudo. Ele é bem bravo, até pra troca ele eu levo um baile com ele.

E: Quais interferências você observou ou observa no desenvolvimento que podem estar associadas a permanência dele na UTI?

R01: Acho que não tem nenhuma, poque eu acho que ele é assim pelo tempo dele e o rim.

E:E como você avalia o atendimento da UTI Neonatal? Tem alguma orietação que você daria aos pais cujo filhos necessitam de internamento?

R01: Olha, para mim o período que ele esteve ali foi muito bom, fomos muito bem tratados, graças a Deus tudo certinho, não tenho o que reclamar, é ótimo o atendimento. E se é realmente necessário para alguém eu não nada do que reclamar, fomos muito bem atendidos graças a Deus.

E: Para finalizarmos, tem mais alguma coisa que queria complementar sobre o Arthur?

R01: Assim, o Arthur é uma criança muito especial para mim sabe, foi uma criança que veio realmente para mudar minha vida, por que eu já não tinha mais criança pequena. Não sei se você sabe, como eu sou uma pessoa de idade e tive ele com 45 anos, daí meu outro filho mais velho já tem mais de 17 anos, então eu já estava só né, ele veio para mudar minha vida, sabe, não foi fácil, nada foi fácil e ainda não está sendo fácil, mas graças a Deus ele tá bem. Não me arrependo de nada, de todo o sacrifício que passei com ele, faria tudo de novo, se fosse preciso, mas ele é maravilhoso, graças a Deus

Uma criança muito esperta, que as vezes eu fico pensando tudo o que a gente passou eu nem acredito que hoje eu tenho ele aqui comigo, como um dia a medica da UTI me chamou pra conversar e a psicóloga mesmo me falou que não me davam nenhuma esperança que ele tava muito mal, aquele dia acabou pra mim, sabe, fiquei muito triste a tarde toda, mas graças a Deus no outro dia eu já tive noticias boas já, que ele não tinha o que tavam pensando que ele tivesse, que era só o rim mesmo, e graças a Deus foi tudo rapidinho, encaminhado para Curitiba, foi uma coisa bem rápida, bem antes do esperado, porque igual elas me falaram, que ia ser demorado, que não era fácil conseguir uma vaga em Curitiba, eu nem esperava que fosse tão rápido, fiquei 15 dias em Beltrão e já fomos encaminhados para lá.

R02

E: Qual a idade deles?

R02: Vão fazer 2 anos dia 28/08

E: Qual o peso e a altura que cada um tem hoje?

R02: Eles estão com 12 quilos, o tamanho não vou saber te falar agora porque não colocaram mais na carteirinha e nas consultas eu esqueço

E: Eles fizeram acompanhamento de Puericultura no postinho de saúde?

R02: Fizeram o primeiro mês só, e depois não foram mais chamados para fazer

E: As vacinas foram feitas certinhas?

R02: Sim sim, as vacinas estão em dia, só falta uma que é de uma campanha q saiu agora e pela gripe ainda não colocaram

E: Mariana, eles possuem alguma doença? ou fazem uso de medicamento de uso contínuo?

R02: Não, nenhum

E: Qual a sua profissão?

R02: Vendedora ambulante e q se fala quando se vende nas casas

E: qual a sua renda mensal mais ou menos?

R02: Em torno 1.200 com mais unhas q estou voltando a fazer tudo depende como vai as coisas

E: Após a alta da UTI Neonatal, como tem sido o crescimento e desenvolvimento dos seus filhos?

R02: Após a alta foi tudo gratificante, eles foram ganhando peso mês após mês sempre os três pesando o mesmo peso. Com 1 ano já estavam querendo caminhar, 1 ano e dois mês já estavam caminhando porém ainda com quase dois anos não estão falando, é o que estou um pouco preocupada no momento, porém penso q pode ser pelo fato de serem prematuro vão pra escolinha, são super queridos tanto em casa como na escolinha, as prof ficam apavorada por eles serem tranquilos.

E: As profes falam sobre o desenvolvimento deles lá? como tem sido?

R02: Sim sim elas comentam bastante que eles se adaptaram com facilidade, não tivemos problema de eles ficarem chorando como acontece, eles amam ir às vezes ficam um dia sem irem já ficam ansiosos pra irem, aí agora nos últimos mês só q eles começaram a

querer brigar entre eles aí a prof falo q no início não eram assim e tal mas como disse elas sempre elogiam eles pelo fato de ser três e serem comportado como são

E: Eles fazem acompanhamento com algum profissional? psicólogo, fisioterapeuta

R02: Não

E: E a compreensão da fala com eles? tipo, quando um adulto fala com eles, como eles reagem?

R02: Sim sim, o Rafael está igual a um papagaio sabe?! querendo falar, mas ainda não consegue tem dias q acho até engraçado ele falando no jeito dele, mas tá falando. O Gabriel reage com tipo “mmm” e com a cabeça ele mostra, ele faz gesto. E o Miguel é mas bravo, a gente fala com ele reage sempre com gesto de bravo tipo resmungando.

Que eles falam e mãe ou mama e pai ou Papai, aí quando a gente pergunta pra eles da Talia que é a mana eles fazem gesto com a mãozinha a onde eles viram que ela foi.

O Gabriel sabe quem é o Rafael e quem é o Miguel. Quando eles estão nos berços fiz a pergunta “onde tá o Rafael?” E ele olhou, e “onde tá o Miguel?” ele olhou pro Miguel

E: Estão bem espertos já...

R02: Sim graças a deus e incrível o desenvolvimento deles

E: Quais interferências foram observadas no desenvolvimento dos seus filhos que você associa com a passagem pela UTI?

R02: O único que eu acho que eles têm a imunidade deles muito baixa, e no meu ponto de vista é por eles serem prematurinhos, mas outras coisas não, o único q trouxeram da UTI foi as chupetas q não consegui tirar até hoje do Miguel e do Rafael, o Gabriel largou por conta. Eles ficam resfriados, melhoram e dali uma semana estão com gripe de novo.

E: E como você avalia o atendimento na UTI Neonatal? Tem alguma orientação que você daria aos pais cujos filhos necessitam de internamento?

R02: Atendimento muito bom, não tenho nem uma reclamação a fazer, nossa, muito bom mesmo. A psicóloga muito querida, a Angela, então eu digo que foi um anjo que apareceu nas nossas vidas, eu estava passando por momentos não muito bom naquele momento e ela me ajudou muito nossa.

Que eu digo quando vejo algum conhecido postar que o bebê está na UTI falo que é um hospital muito bom, que os médicos são muitos atencioso, que fiquem tranquilos que logo vão sair todos feliz como eu saí como meus tri. E tem as psicólogas que mandam fotos e, se a gente precisa de algum apoio elas vem conversar com a gente, nossa, eu fui muito bem acolhida com meus bebês aí nesse hospital.

E: Antes de finalizarmos, gostaria de complementar com mais alguma coisa sobre a UTI ou sobre o desenvolvimento dos bebês?

R02: Desenvolvimento por enquanto só que me preocupa mesmo e eles não falarem ainda, mas que sei que cada criança tem seu tempo né, e as outras coisas, cada dia nós aprendemos com a inteligência deles e da UTI, como disse, nada a reclamar o atendimento deles é muito bom mesmo.

R03

E: Qual a idade deles?

R03: Hoje eles completam 11 meses de nascidos. Na idade corrigida eles teriam 9 meses. Nasceram prematuros de 30 semanas.

E: Qual a altura e o peso deles hoje?

R03: O Gustavo estava com seis quilos e quarenta gramas medindo 63 cm. O Raul estava com 8 quilos e medindo 66 cm.

E: Algum deles apresenta alguma doença? faz uso de alguma medicação de uso contínuo?

R03: Não, doença não. O Gustavo apresentou APLV na UTI, faz uso do Neocat. Não mamam no peito, a pediatra me passou q como o leite não era suficiente era ofertado a mamadeira com as formular aí largaram o peito.

E: Eles fazem acompanhamento de puericultura no postinho de saúde? A vacinação deles está certinha?

R03: Sim fazem, esta sexta tem a próxima vacina.

E: Qual a renda mensal mais ou menos que voces tem?

R03: Meu esposo ganha 1.700, eu trabalho como diarista tiro em média 800 reais por mês

E: Após a alta da UTI Neonatal, como tem sido o crescimento e desenvolvimento de cada um deles?

R03: Após a alta, que fomos pra casa. Eles fazem fisioterapia, O Gustavo que era o menor se desenvolveu bem mais rápido, adaptação foi mais acelerada que a do Raul. O Gustavo já gatinha, ele já fica em pé, ele ficou sentado primeiro, já está nascendo o primeiro dentinho dele, ele é bem ativo, ele é muito esperto e inteligente.

O Raul ele era o maior, mas por ser o maior ele é um pouco mais lento, ele é mais pesado, fica sentado sozinho, ele ainda não gatinha, ele agora ele faz fisioterapia também. Ele quando quer, se ele está sentado ele se estica o máximo que ele pode pra alcançar o brinquedo que ele quer, mas como ele não gatinha ele deita e rola, ele vai rolando até chegar no brinquedo que ele quer e depois ele volta rolando. Mas com os estímulos ele já tá começando a querer sair do lugar, mas ainda não fica de quatro pés, ele ainda não tem o mesmo desenvolvimento que o Gustavo.

E: Eles têm boa compreensão quando falam com eles? tentam falar, se expressar?

R03: Sim, a gente chama eles, eles entendem, eles olham. A palavra não eles já entendem, a gente fala “não pode”, “não, faz dodói”, eles entendem, “pai” eles entendem, “cadê o

papai", são bem compreensíveis assim. E estão tentando falar, eles ficam naquela linguagem deles que a gente não entende nada, uma conversação, uma falação entre eles que é até engraçado.

E: Quais intercorrências foram observadas no desenvolvimento de cada um deles que você associa com a passagem pela UTI?

R03: O Raul não teve intercorrências. O Gustavo fazia apneia nas duas últimas transfusões, transfundiu três vezes. O Raul transfundiu uma vez só

E: Eles precisaram de transfusão devido ao que?

R03: O Gustavo nos dois últimos episódios de apneia, eu lembro bem que a doutora me falou que foi bem difícil fazer ele voltar, principalmente na última, mas as intercorrências com eles seriam essas. Que ele fazia apneia do sono né, e ele transfundiu três vezes, teve infecção duas vezes também, O Raul foi bem tranquilo, ele transfundiu uma vez só, sempre foi mais tranquilo, mais de boa sabe.

Normalmente eles transfundem quando eles tem anemia, eles começam a ficar branquinho, pálido, a corzinha deles começa a ficar meio feinha, eles transfundem, e daí tudo melhora neles, sabe? Eles começam a respirar melhor, eles ficam bem melhor quando eles são transfundidos.

E: Eles transfundiram apenas quando estavam na UTI?

R03: Sim só na UTI

E: Hoje ele não tem mais apneia?

R03: Não, fez episódios apenas na UTI devido a prematuridade

E: Você observa alguma interferência hoje que pode estar associada a estadia na UTI?

R03: Olha até hoje não, São perfeitos. Até eu tinha um medo de que eles pudessem ficar com alguma sequela devido prematuridade, mas não

E: E como você avalia o atendimento na UTI Neonatal? Tem alguma orientação que você daria aos pais cujos filhos necessitam de internamento?

R03: Esqueci de fala, o Gustavo lá por 22 dias de alta voltou pra UTI, Teve q fazer uma cirurgia bilateral de hérnia, elas estavam encarceradas, Aí precisou ficar cinco dias pra recuperar. Então assim, passou, depois que a gente detectou a hérnia ele ficou uns dois dias em casa, eu levei ele consultar e já foi encaminhado pro Hospital Regional, e de mediato a doutora já avaliou ele e já entrou para a sala de cirurgia

Nunca havia entrado em.uma UTI Neo antes. A respeito do atendimento da UTI eu não tenho o que reclamar, eu sempre dizia, tinha uma mãezinha lá que queria levar o filho

dela para a Policlínica, eu olhei para ela e disse “Olha mãezinha, eu tô aqui há 60 dias, com meu filho, eu não tenho queixa nenhuma” É um dos melhores hospitais, os enfermeiros, os atendentes, a pediatra, todo mundo, psicologia, tudo, eu não tenho queixa alguma de nenhum deles, sabe? Atendimento nota 100, se eu pudesse dar outra nota eu daria sabe, por que assim, eles te explicavam, o dia que você não estava bem eles te consolavam, você estava com alguma duvida tentavam te explicar, o que você não sabia elas te ensinavam sabe? É umas pessoas que, de coração, e se tivesse um aqui que falasse “ai vou precisar levar pro Regional’’ eu diria leve, leve que você não vai se arrepender, levem, não esperem. Se tiverem oportunidade, que possam estar dentro daquele hospital, serem atendidos pelos intensivistas, pela pediatria de lá, psicólogos, eu aconselho que levem seus filhos pra lá. Se Deus me deu esses dois pequenos, é porque tinha um propósito, eu conseguia passar por isso.

De momento a gente se assusta né, muito aparelhos, vários leitos, vários bebês, cada um com um caso. Mas fui muito acolhida pelo pessoal da psicologia, pelos enfermeiros, pelos fisio. Com o tempo a gente vai aprendendo a lidar com a situação.

E: Para finalizar, quer complementar com mais algo?

R03: Apenas gratidão, por tudo, porque se não fosse eles meus filhos não estariam aqui. Que os cuidados de uma UTI de pessoas preparadas fazem toda a diferença. Eu não fiquei com ne um trauma sabe? Muito pelo contrário aprendi muito com tudo.

Graças a deus estão bem, crescendo, lógico cada caso é um caso, mas no meu tudo correu deu certo, tudo ficou bem.

R04

E: Qual a idade dela hoje?

R04: Ela tem 2 anos

E: Altura e peso?

R04: Da ultima vez que vimos ela estava com 83 cm e 10 kg

E: Ela possui alguma doença ou faz uso de algum medicamento contínuo?

R04: Ela está há dois anos tomando vitamina e ferro, o biozinco, adivit e neoprofer. O médico receitou até os dois anos de idade.

E: Os exames dela estavam alterados que foi prescrito?

R04: Não, não que eu lembre.

E: Qual seu estado civil? Você está trabalhando atualmente?

R04: Eu sou viúva, recebo o benefício do meu esposo atualmente. Sou formada, técnica em enfermagem, mas eu não estou trabalhando na área atualmente

E: Qual a renda mensal mais ou menos?

R04: Em torno de 2-3 salários

E: Fazem acompanhamento de puericultura no postinho de saúde do bairro?

R04: Sim, foi feito mensal até o primeiro ano dela, agora a gente vai quando precisa, tomas vacinas e tudo mais

E: E a vacinação como está?

R04: Está em dia, tudo certinho.

E: Após a alta da UTI Neonatal, como foi o desenvolvimento da sua filha?

R04: Ela faz acompanhamento com pediatra, nutricionista, fisioterapeuta e fonoaudióloga por um ano, só evoluiu. Conversa quase tudo, caminha, compreende, só não fala certo, por enquanto não vejo atraso, nenhuma sequela. Ainda não vai na escolinha, só em casa, se enturma bem com crianças e adultos.

Quando eu estava grávida eu tive ITU, com 30 semanas piorou, tive sangramento, levou ao nascimento dela, a gente foi encaminhado aqui da cidade para o Hospital Regional, mas não deu tempo de chegar até o hospital, ela nasceu dentro da ambulância do SAMU, ela ficou com a infecção também, nasceu com baixo peso, prematura. Ela entrou no dia 27/07 na UTI e ficou até o dia 10/08, ela só evoluiu lá, tipo, ela precisou de O2 algumas vezes, teve uma PCR.

E: Quais intercorrências foram observadas no desenvolvimento do seu filho devido a utilização da UTI Neonatal?

R04: Acho que a UTI só ajudou ela, no tempo que ficou lá, as pessoas que passaram sempre têm sequelas, não vejo isso, no UCI ela não teve problema nenhum, as pessoas da UTI foram uns anjos, ela teve apoio total da equipe. Não vejo nada que seja referente a UTI

E: Como você avalia o atendimento da UTI Neonatal e quais orientações você daria aos pais cujos filhos necessitem de internamento nessa unidade?

R04: Da equipe, a transparência dos profissionais com o conforto passado, são uns anjos na vida da gente. Terapia maravilhosa, ali estão os anjos que cuidam dos nossos. Pais fiquem tranquilos, ela foi bem assistida, mesmo sem a vaga, porque quando chegamos ainda não tinha vaga na UTI, eles acalmaram. Agradeço a Deus pela experiência.

E: Para finalizarmos, tem mais algo que queira complementar com a nossa pesquisa?

R04: Muitos acham que a UTI é a ultima alternativa, mas não é, é pra quem precisa de ajuda mesmo, de apoio, auxílio, precisam apenas ser assistidas. Pra mim foi bom, eu fiquei bem tranquila em saber que ela ia pra UTI, nesse momento eu soube que ela ficaria bem.

R05

E: Que idade ele tem atualmente?

R05: 1 ano e 8 meses

E: Qual a altura e o peso que ele tem hoje?

R05: Ele está pesando 10,800. Medindo 78cm

E: Certo, ele possui alguma doença ou faz uso contínuo de algum medicamento?

R05: Não, Apenas vitamina e ferro

E: O acompanhamento de puericultura no postinho de saúde, tem sido feito mensal? Como tem sido?

R05: Sim, ele é feito mensalmente.

E: E a vacinação, como está?

R05: Sim, tudo ok

E: Após a alta da UTI, como foi o crescimento e desenvolvimento dele?

R05: Foi super tranquilo, ele nunca foi uma criança que deu trabalho nem nada, eu nunca precisei passar uma noite acordada com ele, nunca precisei é ir pra uma internação em hospital com ele. As únicas doenças que ele teve tipo são no máximo uma febre por causa de algum dente que estava nascendo no início, é ele também teve gripezinhas super levinhas, nossa ele tem um desenvolvimento maravilhoso. Ele sempre aceitou muito bem todos os alimentos, ele ama beterraba, tomate, alface, qualquer tipo de salada ele ama, ele adora, desde pequeno ele mama na mamadeira porque eu não tive leite, ele mama o Neslac hoje, mas ele sempre mamou o NAN. É o primeiro dente dele nasceu com três meses, e ele, nossa, ele tem um desenvolvimento muito bom. Ele já consegue colocar o calçado sozinho, a pediatra falou que isso é uma evolução muito grande, poque crianças de três anos não conseguem, e a hora que a gente fala “ai vamos tomar um banho” ele tira a roupa dele sozinho, nossa ele é muito, muito inteligente, ele fala muita coisa já, ele começou a caminhar com 1 ano e três meses, eu achei que nisso demorou um pouquinho, por que geralmente as crianças começam a caminhar antes né, mas ele demorou um pouquinho para caminhar e também para engatinhar, eu acho que o fato de engatinhar é porque quando ele tava na fase de engatinhar era inverno e eu não gosta de colocar ele no chão, porque tinha medo que ele ficasse doente, pegando frio, mas nisso ele é super tranquilo, é uma criança muito inteligente e se desenvolveu super bem.

E: Quais interferências foram observadas no desenvolvimento do seu filho devido a utilização da UTI Neonatal?

R05: Ele não ter conseguido pegar no peito. Como ele se alimentava pela sonda ele acabou não aprendendo mamar no peito. E quando ele saiu da UTI eu já não tinha mais leite pois já tinham se passado muitos dias então meu leite secou.

E: E como você avalia o atendimento da UTI Neonatal? Quais orientações você daria aos pais cujos filhos necessitam de internamento nesta unidade?

R05: O atendimento que eu dou é uma nota 10, uma nota 100, uma nota 1000. Porque são profissionais muito queridos. A gente tá lá toda desenstabilizada né, e eles sempre tratando a gente com muito, muito carinho, principalmente as psicólogas, as enfermeiras que cuidaram do meu filho, elas tratavam ele com maior amor e carinho, a gente via que realmente elas estão ali sabe pelo amor mesmo, então super, eu fui super bem tratada, eu e meu marido quando a gente ia la visitar, que eu fui um dia tirar leite para tentar dar mama pra ele, a gente sempre foi muito bem tratado ali, então o atendimento foi sempre nota 10. E a orientação que dou para os pais é paciente, porque eles estão cuidando muito bem dos nossos filhos, eles estão fazendo o possível para que quando eles forem pra casa eles não precisem voltar para o hospital. Eu sei que o que a gente mais quer é que a gente não vê a hora que os nossos filhos vão pra casa, mas eles estão fazendo de tudo para que quando eles forem eles não precisem voltar, e que eles vão muito bem pra casa. Então o que eu digo é ter paciência, por que lá eles cuidam muito bem.

R06

E: Que idade ele tem atualmente?

R06: 2 anos

E: Qual a altura e o peso que ele tem hoje?

R06: Agora ele está com 10.800kg e com 86cm

E: Certo, ele possui alguma doença ou faz uso contínuo de algum medicamento?

R06: Não, somente vitaminas

E: Faz acompanhamento de puericultura no postinho de saúde?

R06: Sim.

E: E a vacinação, como está?

R06: Todas em dia

E: Após a alta da UTI, como foi o crescimento e desenvolvimento dele?

R06: Então, o desenvolvimento do Oliver foi tudo dentro da idade corrigida que eles chamam né, que é a idade que corrige com o nascimento prematuro, eles corrigem a idade. Então ele foi tudo dentro daquela idade corrigida né, tudo bem bem certinho as etapas, a gente foi estimulando bastante ele, mas ele foi tudo dentro da idade corrigida. Agora só com dois anos ele está com atraso na fala, mas ele já faz acompanhamento com a fono, faz acompanhamento com fisio também é ainda, pela questão ainda de alguns pontos tipo de andar na ponta do pé, alguma coisinha assim né. E também já faz acompanhamento com psicóloga né, psicologa também trabalha as partes do desenvolvimento dele né, neuropsicomotor né, mas assim a princípio tudo dentro da normalidade até agora, assim só questão mesmo da fala, que é o que tá mais, vamos dizer assim “mais atrasada”, ele fala poucas palavrinhas, mas a questão do desenvolvimento tipo engatinhar, sentar, caminhar, controle né de cervical, de tronco, enfim foi tudo dentro da idade corrigida. Foi tudo bem bem corretinho ali.

Outro ponto também que a gente tá correndo bastante atrás é a questão do peso, ele não não ganha o peso ainda correto que ele precisa estar com dois anos, ele recém tem 10 quilos mas como ele nasceu com 815g então eles falam que tá dentro do previsto assim né, é baixo, bem baixo o peso, mas pelo peso que ele nasceu eles falam que tá tudo bem, vamos dizer assim “dentro da normalidade”.

E: Quais interferências foram observadas no desenvolvimento do seu filho devido a utilização da UTI Neonatal?

R06: Olha, então, eu observo assim, é que até hoje assim ele é muito chorão, ele chora por qualquer coisinha, assim as vezes mínima coisa ele tá chorando; E lá na UTI ele era mesma coisa, então ele chorava muito, muito, muito lá. Na época que nós estávamos lá, as enfermeiras falavam que ele era o mais Chorão, o mais briguento, assim digamos o mais estressadinho de lá de dentro né, e ele é assim até hoje, ele é bastante choroso, nervoso né, até a psicóloga dele acredita que seja da questão desse estresse da UTI ele ficou assim né, assim tipo um pouco mais nervoso, mais choroso, mas eu acho que seria mais esses pontos.

R06: Mas a questão assim, que nem tu pediu antes de doenças essas coisas assim , Graças a Deus assim ele não ficou com nenhuma sequela né, claro, essa questão um pouquinho do atraso né, mas a princípio assim nenhuma sequela, nem pulmonar, enfim, nada, nenhuma nenhuma sequelinha ele ficou, Graças a Deus. Não precisamos, quer dizer, precisamos retornar para o hospital uma vez só para fazer cirurgia de hérnia inguinal, que ele teve de ambos os lados né, mas a princípio foi só para isso, Vamos dizer assim foi tudo bem, bem tranquilo. Outras doenças, outros problemas que tivemos que vamos dizer assim retornar para o Hospital graças a Deus nós não tivemos nada, não precisamos.

E: E como você avalia o atendimento da UTI Neonatal? Quais orientações você daria aos pais cujos filhos necessitam de internamento nesta unidade?

R06: Eu não tenho nenhuma reclamação sobre o atendimento de qualquer profissional lá dentro da UTI todos muitos atenciosos, prestativos, são todos excelentes profissionais, vamos dizer assim dou nota 10 pra todo. A questão de orientação é bem complicado, pois é um momento muito difícil para os pais, mas eu diria pra confiar nos profissionais que eles estão fazendo de tudo e ter fé que tudo vai dar certo. Quando aconteceu conosco, nós não tínhamos ideias de como é esse mundo da prematuridade, então eu diria para os pais também procurarem relatos de outros pais que passaram por isso pra eles verem, entenderem que tudo pode melhorar

R07

E: Que idade ele tem atualmente?

R07: Idade cronológica, 2 anos e 5 meses

E: Qual a altura e o peso que ele tem hoje?

R07: 13,300kg medida 86cm

E: Certo, ele possui alguma doença ou faz uso contínuo de algum medicamento?

R07: Descobrimos recentemente que ela está com asma... está em tratamento de 2 meses depois vamos saber se vai continuar ou não com a medicação, mas a princípio vai fazer uso de bombinha por algum tempo. O principio ativo da bombinha é a Fluticasona e está tomando Singulair um comprimido por noite. Levamos ela no pneumologista em Pato Branco porque ela tosse há vários meses e nada resolve... aí ele receitou esta, faz 15 dias que fomos no pneumologista

E: Faz acompanhamento de puericultura no postinho de saúde?

R07: Fez até os 2 anos de puericultura no postinho. Ela fazia o acompanhamento no CRE quando saímos do Regional, mas logo em seguida ela teve alta. Uma especialidade por vez, a primeira foi fisioterapia, depois nutricionista, daí só continuou com pediatra de alto risco e fonoaudióloga, aí quando ela tinha 1 ano e 4 meses tivemos alta de tudo pois ela já tinha atingido a curva de um nascido a termo e permaneceu.

Também saímos do hospital com o pedido de acompanhamento de neuropediatra, levamos algumas vezes no MACC mas fizemos acompanhamento com dr. Adilson de Pato Branco que fez o Bera (que ela não tinha feito o teste a orelhinha no hospital) e um eletroencefalograma, em seguida deu alta pra ela depois de todos os resultados. A dra do MACC estava pedindo tomografias a cada 6 meses... então consideramos que era muita radiação pra uma criança tão pequena e não fomos mais nas consultas, não íamos discutir com ela... e como tínhamos o parecer de um médico de confiança, desistimos

E: E a vacinação, como está?

R07: Está todas em dia, não teve nenhuma reação de vacina, nem uma febre nada. Sempre fizemos muito uso de medicação caseira, então fazia compressas depois de cada vacina com leite morno, e chegava em casa já dava umas gotinhas de paracetamol, nunca teve nenhuma reação. Única vacina que ela não tomou foi a da gripe deste ano porque como ela está com o pulmão complicado, não melhorou suficientemente pra receber ainda, o restante está tudo em dia, sempre fomos muito cuidadosos com isso.

E: Você trabalha? Qual a renda mensal mais ou menos?

R07: Trabalho, renda mensal em torno de 4 salários entre eu e meu marido, sou casada.

E: Após a alta da UTI, como foi o crescimento e desenvolvimento dele?

R07: Como o de uma criança nascida a termo... não percebemos atrasos

ela gatinhou com 8 meses, andou sozinha com 1 ano e 1 mês

esboçava palavras desde muito cedo mas começou a falar mesmo com 1 ano e meio

agora nos últimos meses ela deslanchou na fala... fala de tudo, pensa por conta própria, tem muita personalidade, dona da própria opinião

ela é incrível kkk

Ficou doente pela primeira vez quando começou ir na creche

o que é bem normal.

E: Quais interferências foram observadas no desenvolvimento do seu filho devido a utilização da UTI Neonatal?

R07: Penso que emocionais talvez

ela tem pânico de médicos, enfermeiros...

inclusive estamos cogitando procurar ajuda psicológica pra ela

desde que saímos do hospital, ela ficou internada 2 vezes apenas, mas ela tem um pânico de ver médicos e enfermeiros chegando perto dela, nem que não seja com ela o assunto kkk

E: E como você avalia o atendimento da UTI Neonatal? Quais orientações você daria aos pais cujos filhos necessitam de internamento nesta unidade?

R07: Nossa, as pessoas são incríveis, como pessoas e como profissionais.

As enfermeiras e as psicólogas muitas vezes me ajudaram a segurar a barra... meu marido era motorista de caminhão na época então eu fiquei quase que o tempo todo sozinha lá com a Tina.

Quanto a orientação, seria mais comportamental mesmo... fé em Deus e confiança nos médicos

quanto menos ficar procurando informações inúteis na internet, melhor pra própria saúde mental

Exemplo, a Tina passou enterocolite 2 vezes enquanto estava na UTI, meu marido, longe, ficava pesquisando sobre a doença, lendo, então pra ele não enlouquecer na estrada e não me deixar pirada também, muita coisa do que acontecia na UTI eu guardava pra mim e nem comentava com ele nem com ninguém, pra não surtar. A primeira vez ela ficou 7 dias em jejum... na segunda 10

12 dias de antibióticos, PCR não baixava na segunda vez, aí que foram descobrir que era o acesso muito antigo que estava interferindo no PCR, mas as pessoas me perguntavam como ela estava, eu dizia que estava tudo bem, o que eu menos queria era ter que ficar dando explicações.
Olha, a gente tem que ser forte, pela gente e por eles.

E: Antes de encerrarmos, tem mais alguma coisa que queira compartilhar comigo sobre sua experiência com UTI e desenvolvimento infantil da sua filha?

R07: A Valentina é um presente de Deus, um verdadeiro milagre. Eu fiz todo meu pré natal com médico particular, fizemos exames e tudo que era necessário e ele não viu que minha placenta e cordão umbilical eram insuficientes, o cordão era mais fino que um dedo mindinho e a placenta media o tamanho da palma da mão, foi pra biópsia, não tomei corticoides porque ele não me receitou, e ainda assim ela nasceu com 94% de saturação, com 1,145kg e 38cm. Fui pro SUS porque fiquei sem líquido e quando o médico viu disse que não poderia mais fazer nada, que eu estava por conta própria porque ele não era parte de nenhum corpo clinico, se hoje eu engravidasse novamente com certeza faria todo o pré natal no SUS.
Valentina fez uso de O_2 por 2 dias apenas na Neo, ela é muito ativa e esperta, mais inteligente que o pai dela e eu juntos, arteira, como toda criança saudável graças a Deus.

R08

E: Qual a idade dela?

R08: Ela tem 2 anos e 2 meses

E: Qual a altura e o peso que ele tem hoje?

R08: A ultima vez que pesei ela estava com 11,900kg

E: Ela tem alguma doença ou faz uso continuado de alguma medicação?

R08: Não, não faz uso de nenhum medicamento. Ela toma vitamina né, só para complementar mesmo. A gente levou ela no oftalmologista, neurologista, tudo liberado, não tem nada.

E: Como tem sido o acompanhamento dela? Faz no postinho de saúde? Como tem sido?

R08: Então eu faço pelo Cras que é em Beltrão. E faço com o Dr Mauro que é particular. Eu levo no posto para as vacinas. Neurologista, oftalmologista foi no particular, uma vez eu levei ela pelo SUS neurologista.

E: Vacinas todas certinhas então, em dia?

R08: Sempre

E: Qual a sua profissão?

R08: Cabelereira e manicure, faço maquiagens e vários procedimentos.

E: A renda mensal é mais ou menos de quantos salários?

R08: Tipo o meu salário tem mês que dá bem, tem mês que não, depende do mês da 3.

E: Após a alta da UTI, como foi o crescimento e desenvolvimento dele?

R08: Oi bom dia tudo bem? Então você me fez umas perguntas ali, é quando ela veio da UTI, foi assim, ela ganha peso tipo não tão rápido né, como uma criança que nasce de 9 meses, é mais devagar né mas ela assim é muito espertinha assim. Começou a andar com 9 meses, tipo ela nem gatinhou, ela já começou a andar pelo sofá, dar os passinhos e foi assim muito rápido. Só na fala assim que ela tá demorando mais um pouquinho né, ela demora mais para falar ta demorando mais pra falar, mas ela fala "mamãe", "papai", "neném", o "au-au", tem um cachorro que se chama "Toddy" ela fala super bem Toddy. Então assim a gente ajuda bastante ela também né, mas assim, na fala assim achei um pouco meio demorado, e nos dentinhos, ela tá com pouquinho dentinho na boca ainda né, mas o resto tudo certo. Tipo quando ela veio para casa, a gente começou a dar banho no chuveiro né, o pai dela segurando ela e dando banho no chuveiro, isso foi muito bom, assim sabe, para o desenvolvimento dela achei que foi muito bom

E: Quais interferências foram observadas no desenvolvimento do seu filho devido a utilização da UTI Neonatal?

R08: Tipo na verdade, eu não estava preparada né para essa situação, de ter um bebê daquele tamanho né, que era muito pequenininha, demais, e eu não estava assim preparada para ser mãe de UTI sabe. Eu nem sabia que existia, que poderia existir isso, de uma criança nascer com esse tamanho né, eu sabia que nasciam prematuros, não prematuros extremos né, que ela que o caso dela foi 24 semanas no caso por ali, 25 semanas. Então foi uma experiência assim ruim e boa, eu amadureci muito né, eu já vejo as coisas bem diferentes. E outra, eu achava também que ela ia chegar e ia ter um pouco de dificuldade, porque ela foi entubada né, tomou muitos medicamentos, eu achei que os dentinhos dela ia nascer meio já com cáries né assim porque tomou muito antibiótico essas coisas, mas não, não, não, isso não mudou nada, nada. Até parece que ela foi uma criança que nunca ficou lá na UTI assim, uma criança normal que nasceu pequenininha, que veio para casa, normal, nunca se afogou com leite, com nada, mamou no peito por pouco tempo, porque a gente pegou o COVID né daí acho que o medicamento tipo interferiu no sabor do leite, eu acho que seja isso. Ela parou, daí agora ela toma o NAN, a gente dá um leite bem bom para ela né, ela come, pouco mas ela come.

E: E como você avalia o atendimento da UTI Neonatal? Quais orientações você daria aos pais cujos filhos necessitam de internamento nesta unidade?

R08: Você me perguntou ali, nossa, o atendimento sabe foi maravilhoso, não tem o que agradecer a equipe médica né, as enfermeiras, técnicos de enfermagem, tudo, Meu Deus, não tem explicação, a psicóloga Ângela meu eu amo ela de paixão, todas elas né. A Ângela passa uma calmaria para gente assim que não tem explicação, tipo assim quando eu tava desesperada com dor no coração parece que eu falava com ela parece ser era tipo um remedinho assim, aí eu vinha para casa nos finais de semana daí eu falava para o meu marido, "ai eu preciso voltar lá porque eu preciso ver a Ângela, eu preciso do meu remédio, eu preciso de um remedinho", que era a Angela assim, que ela, ela me passava muito carinho, muito amor, que eu precisava demais, sabe?! eu precisava, eu precisava dela, assim ela não podia pegar folga assim sabe, as vezes ela falava "Ai simone eu to de folga, eu preciso pegar folga também, eu preciso descansar", mas assim eu não aceitava sabe, ela tinha que ta ali, a hora que eu chegasse ela tinha que ta ali pra ela me acalmar. Ela me acalmava muito assim, as outras também mas era a angela né, ela me passa tipo que ia ficar tudo bem, que ia dar tudo certo.

Eu sempre falo assim sabe, que as mãezinhas ali tem que ter mais atenção, claro que os bebe também ne, mas as mãezinhas ali meu Deus assim, tinha que ter mais atenção assim, porque é uma coisa assim que não tem explicação, um sofrimento muito grande, dói demais. Ao mesmo tempo alegria, ao mesmo tempo tristeza, ao mesmo tempo é um medo assim que vem, assim que chega, assim que não, ai sabe, é uma coisa muito louca, é uma experiência muito louca assim sabe. Hoje eu fico pensando né, Meu Deus o que eu passei foi bom para mim né, para minha vida assim, foi bom e foi ruim também, sei lá, é uma coisa é uma emoção muito grande, uma coisa muito louca sabe, uma coisa louca, louca, não tem explicação para essa situação.

E assim é, e o negócio assim das visitas sabe?! Tipo, as mãezinhas era muito bom assim, as visita, as mãezinhas lá dentro da UTI né, que faz muito bem para as crianças, faz bem para elas também, elas ta acompanhando porque elas sentem que tá ajudando, eu assim quando eu vinha para casa eu achava assim que eu abandonava a neném lá e a neném ia passar mal tipo, a neném vai passar mal se eu não tô lá, e se eu não tava lá não precisava nem tocar na minha neném, mas assim eu senti assim que eu tava dando força para ela, tava dando energia boa para ela, sabe?! então assim as visitas das mãezinhas é muito bom, é ótimo, mas as visitas dos avós, dos tios, dos primos, das madrinhas ali eu acho que não seria ótimo porque nem todo mundo né, às vezes está com alguma coisinha mas não fala para poder curiosar para poder olhar o bebê lá. Eu acho assim que seria bom só a mãezinha mesmo

Porque quando eu tava lá vinha gente de vários lugares, e atendia telefone lá dentro da UTI, sabe?! essas coisas assim é bem complicado né?! Porque as pessoas não têm noção né, nem todo mundo tem uma noção, Sabe?! Tem gente que não, a mãe ela sempre vai se cuidar porque ela quer o melhor para os filhos dela né, mas os outros assim não né, não sei se essa questão que você me perguntou ali, se eu entendi direito, eu acho assim que era bom só a mãe e o pai, o resto assim eu acho que não poderia ser liberado dentro da UTI, porque deve, porque nessa história aí dessas doenças que aconteceu aí, tipo eles proibiram um pouco né, as visitas e eu acho assim que, que foi bom assim, para Helena, foi ótimo pra Helena, que daí pelo menos não pegou nenhum tipo de bactéria nada né correu tudo bem

R09

E: Qual a idade dele?

R09: 2 anos completos

E: Qual a altura e o peso que ele tem hoje?

R09: Ele tá em torno de 12kg e a altura tem em torno de 80cm

E: Ela tem alguma doença ou faz uso continuado de alguma medicação?

R09: Ele estava com bronquite, ele pegou várias gripes que foi acumulando e virou uma bronquite. A gente está tratando essa bronquite dele. Ele toma sulfato ferroso e adtil que elas pedem para dar até os dois anos, agora ele vai parar.

E: Como tem sido o acompanhamento dela? Faz no postinho de saúde? Como tem sido?

R09: Ele faz acompanhamento no 18h com a medica pediatra, desde que ele nasceu. Não fez outro acompanhamento, ele não precisou fazer nenhum outro, de nada. Não foi feito o acompanhamento de puericultura

E: Vacinas todas certinhas então, em dia?

R09: Está em dia.

E: Qual a sua profissão?

R09: Carteira assinada, sou gerente, fazem 3 meses

E: A renda mensal é mais ou menos de quantos salários?

R09: Em torno de 2 salário contando com a comissão.

E: Após a alta da UTI, como foi o crescimento e desenvolvimento dele?

R09: Foi bem tranquilo assim, na verdade o que foi difícil foi a primeira semana que ele saiu do hospital, porque é, como ele ficou 27 dias internado eu não, ele não mamava assim com força no peito, então eu nunca tive muito leite assim, eu tinha mas não tinha muito então assim ele sempre tomou complemento, sempre, sempre, sempre, ele nunca ficou sem tomar o complemento, então ele mamava no peito, mas mamava um pouquinho do complemento depois, daí na primeira semana ele saiu do hospital ele tava com 2 quilos e 60, na primeira semana ele já foi pra 1,900 e pouco, porque daí ele não mamava direito no peito, porque ele só dormia gente, imagina ele era prematuro, ele só dormia, pra acordar ele era difícil, pra acordar ele eu tinha que passar lenço umedecido na cara dele, nem assim ele acordava. Daí não mamava muito, assim só queria dormir, dormir, dormir, daí eu, e eu sofrendo tentando dar o complemento no copinho que nem elas pediram pra não dar confusão de bico nem nada, Daí eu vi que ele tava, daí eu levei ele, aí quando ele

ganhou alta, ele consulta a cada três dias né logo que ganha alta, daí depois que passa a ser 15 dias, depois 1 mês e assim vai indo. E aí na primeira semana ele baixou o peso a doutor ficou preocupada falou "ó se ele não engordar ele vai ter que voltar pra sonda", daí eu fiquei meio desesperada né. Daí eu fui lá na farmácia e comprei a menor mamadeira que tinha que parecia assim de boneca mesmo de tão pequenininha que era, e aí eu comecei a dar o suplemento na mamadeirinha pra ele, daí ele tomava bem daí ele começou a ganhar o peso. Mas ele mamou no peito até os 6 meses, daí depois ele não quis mais, depois dos 6 meses, daí eu tentei, fiquei 3 dias insistindo e ele não queria mais.

Ele é um tagarela, ele fala tudo, ele já conversa por frases. Eu fiquei em casa com ele até um ano, até ele fazer um ano eu fiquei em casa com ele. Parei de trabalhar para poder justamente acompanhar esse desenvolvimento dele né, e eu fiquei em casa com ele, ele fez tudo no tempo certinho. Ele não, fora essa primeira semana logo depois que ele ganhou alta e que demorou um pouquinho para engordar, depois foi tudo bem certinho: ele mamou até os seis meses, no dia que ele completou oito meses ele começou a engatinhar de verdade, ele começou a caminhar quando ele tinha um ano e pouquinho, um ano e uns vinte dias mais ou menos, e aí depois ele sempre, foi todos os sinais né, os sinais de prontidão, quando a gente vai dar comida, tudo, ele fez tudo no tempo certinho.

E: Quais interferências foram observadas no desenvolvimento do seu filho devido a utilização da UTI Neonatal?

R09: Eu acho que, eu acho que nada de ruim assim, eu acho que uma coisa boa que ficou da UTI foi a rotina que ele tinha lá, e eu acho que ele aprendeu também a ficar sozinho na incubadora, porque não podia pegar né, então ele sempre dormiu meio que sozinho, eu nunca precisei embalar muito ele, eu acho que nada assim que prejudicou o desenvolvimento dele.

E: E como você avalia o atendimento da UTI Neonatal? Quais orientações você daria aos pais cujos filhos necessitam de internamento nesta unidade?

R09: Nossa eu achei perfeito gente, é tudo assim ó, o atendimento deles é sensacional, os profissionais são todos ótimos, as psicólogas, os médicos, os enfermeiros, todo mundo, eles realmente cuidam assim, como se fossem deles sabe?! E eu assim, nossa eu não tenho nada, zero, zero reclamação, não tenho nada pra reclamar, foi tudo muito bom, a gente foi muito bem atendido, eu também quando fiquei internada lá, precisei, eu tava com pressão alta e fiquei uns 5 dias internada, fui muito bem atendida, o Levi, meu Deus, foi muito, muito bom, eu sinto assim ó, fico muito agradecida até hoje pelo pessoal, com tudo o que eles fizeram e eu acho que é bem difícil assim, pra gente, pros pais, esse período

que a gente fica lá, então aquela salinha que elas tem para as mães é bem importante por que ali a gente divide as experiências, a gente conversa, uma consola a outra, é bem bacana aquele espaço que eles tem ali para as mães ficarem, e eu acho que os pais tem que unir força, e tem que ter bastante paciência por que passa, a fase da UTI passa e depois tem muitas alegrias...

E: Tem mais alguma coisa que queria compartilhar sobre o desenvolvimento do seu filho?

R09: Acho que foi tudo muito bom, muito bem a evolução dele, a ajuda dos profissionais, a assistência do hospital, acho que foi tudo perfeito, assim não tenho nada a reclamar, assim, nada, só elogios mesmo e depois o acompanhamento que a gente tá fazendo lá no 18h, que eles encaminham d

R10

E: Qual a idade dele?

R10: 2 anos + 2 meses

E: Qual a altura e o peso que ele tem hoje?

R10: 11,100kg, medindo 84cm

E: Ela tem alguma doença ou faz uso continuado de alguma medicação?

R10: Ele nasceu com complicações respiratórias bem graves e com um rim só. Ele tem resistência a antibiótico, bronquite e usa bombinha, tem imunidade muito baixa, fica doente com recorrência. Seretide + montelucaste + polivitamínicos+ Kaloba + desloratadina

Tem infecções recorrentes.

Acredito que por ele ter usado muito antibiótico na UTI, seja mais difícil pra ele um antibiótico mais simples fazer efeito.

E: Como tem sido o acompanhamento dela? Faz no postinho de saúde? Como tem sido?

R10: Sim, desde que ele nasceu ele faz. A gente tem uma pediatra aqui no município, ela acompanha desde que nasceu. Fez com neurologista, está encaminhado ao otorrinolaringologista. Mas no postinho também foi feito, tá tudo na carteirinha.

E: Vacinas todas certinhas então, em dia?

R10: Está em dia.

E: Qual a sua profissão?

R10: Concursada, no município

E: A renda mensal é mais ou menos de quantos salários?

R10: 2 salários

E: Após a alta da UTI, como foi o crescimento e desenvolvimento dele?

R10: Foi assim, bem, os primeiros 90 dias foram bem difíceis assim, bem difíceis assim, porque ele tinha, eu tinha, eu tenho um momento até hoje né, e a gente tentou amamentar desde que o momento que ele saiu da UTI que ele chegou no meu colo eu já comecei a amamentar, mas como ele era ele perdeu bastante peso na UTI então foi me orientado que ele tinha que entrar com uma com leite artificial né. Isso foi bem difícil, porque ele não queria mais o peito, ele queria só a mamadeira né, e ele tinha muita cólica, muita cólica, ele veio de lá com muita cólica, daí nos primeiros, ah eu não vou, ah isso eu esqueci, ele foi pro neuro também, quando a gente veio ele foi com a doutora Greice, porque ele teve

convulsões logo que ele, logo que a gente veio da UTI, ele teve vários episódios de convulsão... depois da alta, em casa. E daí assim, foi a muito, ele chorava muito, daí ele se batia assim, nossa foi bem difícil, os primeiros 40 – 90 dias a gente não dormiu nem uma noite duas horas, nunca. E ele assim, ele ganhou bem pouquinho peso nos primeiros 60 dias, depois dos 60 dias que a gente decidiu que se ele largasse o peito a gente ia deixar, ele precisava mamar né, não podia ficar é proibindo ele de mamar a mamadeira, insistindo no peito porque ele não ganhava peso, aí a gente liberou a mamadeira em mais horários né, aí ele pegou o peito. Aí com 90 dias ele pegou o peito normal, daí ele, ele fez opção de mamar nos dois numa boa, uma hora ele mamava na mamadeira, outra hora ele mamava o peito e bem, daí dos 90 dias em diante sim, ele começou a ganhar peso, bastante peso por mês e foi tudo, daí foi tudo normal, tudo bem tranquilo, mas os primeiros 90 dias foram bem tensos, bem difíceis, bem complicado, umas noites. E o estresse mesmo né, de o medo de... ele precisava do leite do peito e ele não, ele não aceitava, ele queria só a mamadeira, trocamos uma, duas, três mamadeiras, tentamos dar no copinho os primeiros dias, mas daí como ele se afogava, ficou bem difícil com o copinho, então com a mamadeira saía menos, era mais fácil. Tentamos com a seringa, nós fizemos muitas coisas nos primeiros dias, muitas tentativas. Aí ele ia praticamente toda semana com a pediatra, relatava tudo pra ela, ela dizia “não vamos tentar isso agora, agora vamos tentar isso” e eu tinha graças a Deus o telefone dela no meu, então qualquer coisa que acontecia eu pedia socorro pra ela, ela sempre me respondia. Então assim, foram bem, bem complicados, mas com 90, os primeiros 90 dias assim foram muito tensos, depois a Dra Greice falou pra mim que provavelmente as convulsões eram pelas medicações da UTI, aí assim que passou essa era uma coisa que ele tava eliminando do organismo, a hora que passasse não ia dar mais, e realmente não deu mais, aí com 90 dias ele começou a regular o sono, seus horários, e as mamadas mais longas, e daí assim, aí foi um respiro. Mas ele não dorme bem até hoje... Não, não dorme bem até hoje, se bate, acorda bastante e ele se bate bastante durante a noite, ele, assim, ele é bem, ele é uma criança super calma durante o dia, eu sempre falo “o Joao me dá menos trabalho durante o dia, do que durante a noite dormindo”. Ele se bate bastante, eu tava, eu já falei umas quantas vezes isso pra doutora, ela falou pra mim que ela acredita que seja pela respiração, daí então, por isso a gente vai voltar agora com o Marcio pra ver se não ficou alguma sequelinha dentro por que ele tem sempre o nariz trancado, sempre, e ele ronca, então a gente vai voltar pra fazer um, mas assim ele, ah eu dormir uma noite, eu vou te falar a verdade a única noite que eu dormi uma noite inteira ele tinha 6 meses, eu e a minha menina cuidávamos e revezávamos

durante a noite, nós dormimos uma noite inteira, assim tipo ele dormiu 10 horas e era 7h da manha e ele não tinha acordado, daí quando eu acordei 6 horas eu me desesperei, me deu um ataque de nervo, botei a mao ver se ele tava respirando escutei o coração, tentei acordar ele dormindo pensei meu Deus, meu Deus o que que eu fiz eu dormi a noite inteira e não vi, mas ele dormiu, ele tinha dormido, ele tava bem então pra mim assim foi desesperador ver ele dormir tantas horas assim sem se bater, dormiu super bem, uma noite quando ele tinha 6 meses, e assim muito lá de vez em quando ele consegue dormir 4h na madrugada assim sem acordar. A doutora também acha que é isso (bronquite), ele não aceita um travesseiro mais alto, que se ele conseguisse dormir num travesseiro mais alto, de repente ele conseguisse respirar melhor e dormisse melhor né, mas ele não aceita, ele não quer ele não quer travesseiro, ele dorme direto no colchão, aí se bota travesseiro ele vai rolando, rolando, rolando até ele tirar o travesseiro. Ele não gosta, ele dorme no colchão, e ele tem dois dos "titi", que nem diz ele que é do Mickey, então ele tira um coloca o outro né, e eu sempre passo óleo no travesseiro que daí fica o cheirinho pra ele dormir melhor, mas ele não, não gosta, ele gosta de dormir direto no colchão, retinho. E daí ele tampa o nariz né. E ele é muito, com muita personalidade, muita, muita personalidade, ele é muito, eu acho, eu sempre me preocupo por que ele é um bebe de dois anos, mas ele é, e ele tem criança, ele tem contato todos os dias e agora na creche e tudo né, e mesmo aqui em casa eu sou rodeada de criança ao redor da casa, mas ele é muito, muito adulto. Ele é muito pra frente do tempo dele, ele tem dois anos, ele sabe todas as cores, ele sabe todas as cores, ele reconhece os numero até o 5, e ele, ele assim reconhece o nome dele em qualquer lugar e ele foi pra creche esse ano, ele nunca tinha ido pra creche, e ele nem, eu nem posso dizer assim que é uma frequencia direta porque agora ele ficou 15 dias em casa, que ele tava com infecção, e em casa eu não sou aquela mae louca que fica ali tentando deixar, fazer a criança crescer antes do tempo, não, os brinquedos dele são todos direcionados, tipo, ele tem brinquedos educativos, mas ele, ele tem uma memória fotográfica que é uma coisa impressionante, impressionante. Se você for com ele num lugar de carro, quando tu passar próxima vez ele vai saber, ele vai saber, ele guarda, eu sou péssima, péssima nisso, eu não guardo nome, lugares, rostos né, não, ele é impressionante, assim impressionante, tem uma memória incrível. Hoje a profe falou pra mim "Mãezinha, o joao não vai chegar nos 4 anos sem ler, ele reconhece o nome dele aonde estiver escrito", isso é uma coisa que eu não ensinei, 2 anos, eu não ensinei nada disso pra ele, tipo ele aprendeu o nome dele, lá na escola tem o quadradinho " esse é do joao, esse quadradinho é do joao, tá então é do joao", mas ele, ele gravou na memoria

fotográfica dele o nome dele como que escreve, então aonde ele ver o nome dele, ele reconhece pela memoria, pela foto, porque ele não tem compreensão de leitura né, então, é pela memória fotográfica que ele reconhece o nome dele. Ele é muito inteligente.

Ele demorou bastante para falar, ele demorou bastante, pra falar. ele caminhou com um ano, um ano e dois meses, ele caminhou, normal, tudo dentro do normal. Tipo com 11 meses ele tava caminhando assim, se soltando já, aí eu fui com a pediatra, a pediatra falou pra mim, nós começamos a perceber que ele tava com um pezinho virado, né tipo assim um pezinho tava sempre virado, aí eu, a doutora falou pra mim "filma ele caminhando pra mim", dai eu falei pra ela, ela falou pra mim " agora aqui ta normal né" netao ela falou, "então quando ele pegar o.." eu tinha aquele andador de empurrar, naquele ele andava por tudo, né, aí ele pegou, quando eu fui filmar ele, eu filmei eu mandei pra ela, ela falou "Olha Edila, q perninha dele não tá preparada ainda, então não insiste mais, ele levantou por conta dele tudo bem, mas vocês não insistam mais ele pra caminhar porque a perninha dele ainda não tá pronta, só a direita" aí a gente parou, deu uma diminuída assim na insistência pra ele caminhar e ele levou mais três meses, daí com mais três meses sim, aí ele caminhou com a perna retinha certinha como tinha que ser. Ele engatinhou normal, ele engatinhou com 6 pra 7 meses ele engatinhou, de quatro antes ele se arrastava, daí de 4, ele gatinhou. A parte motora dele foi super tranquila. O falar demorou um pouquinho mais, e ele ainda fala bem enroladinho, assim, ele é bem bebê, ele fala bem, bem, ele fala bem diz a minha filha que ele fala árabe, a gente intende né, a língua dele, fala bem bebê. Isso pra nós é novidade porque o Lucas e a Julia, eu tenho dois, o Lucas e Julia sempre falaram tudo, meu neto também assim, eu tenho um neto também, seis meses mais novo que o Joao, ele também fala perfeito, perfeito, igualzinho a Julia e o Lucas, ele fala tudo correto, tudo certinho, o Joao fala tudo que nem bebê. Uma outra coisa que a prof também me pediu hoje, ela me pediu hoje se a gente cuidava assim em casa eu falei "Capaz profe", falei " a gente fala do jeito que dá, tudo meio errado" falei, "a gente não tem esse cuidado", porque ele, tipo ele configura todos os plurais, se são dois ele, por exemplo eu boto os pratos na mesa pras crianças comer ele fala "um, dois pratos", ele corrige tudo com o plural, se são dois são dois pratos, dois pratos ele fala pra ela, dai ela falou "Edila, ele tipo, não é dois prato, é dois pratos", é tipo, fala errado mas com o plural. E ela falou isso e eu falei "Mas capaz, profe" falei " Daonde que a gente" falei "ele pode ter visto na TV, nos deseinhos ali do joaozinho que ele assiste, mas nós não" então, mas é. É na verdade a parte intelecto dele me da até um pouco de medo, por que eu acho que ele é a frente, sempre a frente do tempo dele, sempre a frente, eu vejo porque são seis

meses de diferença com meu neto, que também é muito estimulado lá por eles e tem a maninha tudo em casa, mas nossa, o meu neto é bem bebê, bem bebezinho, assim, não tem, nossa, o joao perto dele é parece que tem quatro anos por ai. Algumas coisas que ele faz que eu digo pra Julia “Julia, você tem dezessete e você não enxerga as coisas como o teu irmão com dois enxerga. Assim, coisas assim, de sensibilidade, por exemplo, chegou as pessoas aqui em casa ele tem preocupação como se ele fosse um adulto mesmo, isso me preocupa por que eu acho que não é pra ele né, não é a idade dele né. Todo mundo tem que tá sentado, tipo, se entrou ele já busca uma cadeira, não pode ficar de pé no chão nunca, porque ele busca um chinelo, bota no pé uma pantufa, tem que calçar o calçado, sim, sentou as pessoas no sofá, tamo conversando daqui um pouquinho ele já vai abrir o balcão vai lá trazer uma bolacha, ele vai trazer, ele se preocupa como se ele fosse o dono da casa. Eu me deixa preocupada por que eu acho que ele tinha que ser mais bebe né, dois anos né, essa compreensão dele isso vai pesar pra ele em algum momento né, vai pesar, agora tudo bem ne´, mas assim em algum momento vai pesar pra ele né porque ele ta além do tempo dele. Penso assim que quando ele for pra escola, não to sofrendo por antecedência né, mas por exemplo na escola como a profe mesmo disse, ele relata tudo pra profe o que que tem que fazer, então, a profe vai trocar ele fala pra ela “o lenço, pega o lenço, prof, passa no nenê, agora pega o creme, agora passa pomada, na bundinha do nenê, agora a fralda”. Tudo, eu falo pra Julia que ele tem TOC, porque tipo eu não posso mudar as coisas de lugar, tem que tá sempre no mesmo lugar por que ele sabe que é ali, ele tipo se você pergunta pra ele qualquer coisa de dentro de casa assim, ele já sabe que é ali que ta, então se você não pode trocar as coisas de lugar porque ele enloca, se encomoda, ele vai lá e guarda no mesmo lugar. Tu chegou o calçado ele tem que levar lá pra lavanderia, não pode deixar na porta, não pode deixar o calçado virado, ele parece um velho, ele parece um velhinho, velhinho da mae, cuidador, é bem assim mesmo. É bem diferente mesmo, é por isso que eu fico bem, deus me livre se falar pra ele que você tem um dodói ele saiu moendo, vai la no quarto pega a pomada dele, cura tudo, a pomada dele de assadura cura tudo, ele vai lá e ele te enche de pomada e ele faz massagem, e assim, ele é um amado. Na verdade, ele é um presente de Deus, eu falo isso todos os dias, ele é um milagre de tá vivo, de ter vindo nessa idade que eu to, ele é maravilhoso, e o que eu tenho de gratidão assim pelo hospital regional pelo que fizeram por mim ali, porque o joao foi muito difícil, ele teve muitas complicações dentro da Uti, e ele sair de lá assim perfeito como ele é, é porque era pra ser de Deus mesmo. Não tem outra explicação.

E: Quais interferências foram observadas no desenvolvimento do seu filho devido a utilização da UTI Neonatal?

R10: Na verdade, agora hoje, pensando assim o que ele teve, acredito assim que essa questão do sono né, de repente seja ainda uma consequência pela medicação, por ter sido sedado por tantos dias né. Por ele não conseguir dormir e a gente acredita realmente que o problema ali no canal seja também uma consequências da intubação né, que tenha ficado uma lesãozinha alguma coisa porque chegar a fazer um barulho do lado esquerdo assim dele do nariz, da parte interna, mas agora vamos fazer, o Dr vai pedir uns exames com certeza né, assim que eu conseguir levar pro otorrino, e daí a gente vai ver por lá, mas a gente acredita que possa ser uma consequência da intubação, que não é uma coisa anormal em criança que são entubadas né. A ronqueira, do nariz trancado. Porque ele já tinha isso quando a gente veio de lá, daí o doutor falou "a gente não vai judiar dele agora, pra fazer uma vídeo né, por que ele tá tao pequenininho, tao bem, vamos cuidar e mais pra frente quando você voltar a gente volta". Agora o doutor identificou que realmente pode ser uma consequência da intubação. Mas isso vai ser uma descoberta dos próximos dias, assim que a gente fazer os exames e levar ele.

E: E como você avalia o atendimento da UTI Neonatal? Quais orientações você daria aos pais cujos filhos necessitam de internamento nesta unidade?

R10: A palavra do atendimento da UTI é gratidão, nós fizemos uma lembrança pra levar para todas as profissionais, 60 e poucas lembrancinhas, pra levar pra elas, e eu tenho elas até aqui em casa até hoje, porque quando a gente veio a gente iria retornar ali no regional, eu fiz uma vez a gente não conseguiu ir, fiz outra vez não consegui, daí ficou muito forte a pandemia, aí a gente não saiu mais com ele. Entao eu tenho elas guardadas até hoje aqui em casa, agora quando eu retornar, que agora graças a deus ele tá maior, ele ta mais tranquilo, agora quando eu retornar com o Dr Marcio lá, a gente vai na UTI levar.

A gente queria que tivesse o nosso cuidados e a nossa gratidão ao que é aquele cuidado dentro da UTI, e assim, de uma forma muito especial, a Dr carol que é uma benção, assim, meu Deus pra mim ela era uma referencia de libertação porque toda vez que eu via que ela ligava que eu atendia o telefone meu coração dava uma aliviada e a Angela e a Sabrina nem se fala. Elas duas é, na verdade todas mas essas são as que a gente tem mais contato, né. Mas elas são seres de luz, eu digo pra elas sempre, são seres de luz. Sempre, sempre, sempre muito bem atendidos. O suporte que dá, as pessoas nem sabem que isso existe né, a gente sempre se identifica quando tem um pai de UTI eu até hoje, porque lá eu to sempre mandando os pais lá pra visitar as crianças quando tao na UTI, as mães pra levar o leite,

então a gente sempre troca uma ideia lá pelo vidro do agendamento, toda vez que tem um bebe que vai pra lá e que os pais precisam ir que a gente transporta, então a gente sempre tem essa troca né, e é sempre a mesma fala.

R11

E: Qual a idade dela?

R11: 3 anos

E: Qual a altura e o peso que ela tem hoje?

R11: 15 kg

E: Ela tem alguma doença ou faz uso continuado de alguma medicação?

R11: Não, nada

E: Como tem sido o acompanhamento dela? Faz no postinho de saúde? Como tem sido?

R11: Logo depois que ela deu alta a gente teve que fazer acompanhamento no Regional, aí fazia com os médicos, tipo com neurologista teve que fazer, daí a gente fazia acompanhamento com fisioterapeuta aqui onde nós moramos. E a gente fazia puericultura mas não fazia no posto de saúde, daí eu fazia particular no caso com o médico daqui.

Aí no regional a gente foi uns dois meses só depois que ela nasceu que daí conforme ela ia indo nos especialistas ela ia ganhando alta de todos e a gente nao precisava mais retornar.

E: Vacinas todas certinhas então, em dia?

R11: Sim, em dia

E: Qual a sua profissão?

R11: Professora

E: A renda mensal é mais ou menos de quantos salários?

R11: Acho que 4 mais ou menos

E: Após a alta da UTI, como foi o crescimento e desenvolvimento dela?

R11: Foi tudo muito tranquilo assim, depois que ela deu alta na UTI a gente ficou acho que três dias só na enfermaria daí gente vem para casa. Daí, já a fisioterapeuta começou de ir em casa fazer fisioterapia, mas ele mesmo falava que não parecia que era uma criança prematura assim. Mas ela, nossa foi bem, bem tranquila mesmo, nunca teve nada assim, de problema, de dificuldade, nenhuma.

Não, começou tudo na época certa assim: falar, como eu tenho uma outra que é mais velha, ela tipo demorou um pouquinho mais que a outra né, mas ela falou antes dos dois anos ainda. Que eu sempre falava que, que eu sou muito preocupada, meia histérica eu acho, eu falei que se até os dois anos ela não começasse a falar a gente ia procurar uma

fono e ver né o que que tava, mas assim parece que foi, faltava tipo uma semana para dois aninhos aí ela tava falando de tudo já, então acho foi bem, tudo na época certinho.

E: Quais interferências foram observadas no desenvolvimento do seu filho devido a utilização da UTI Neonatal?

R11: Na verdade assim, é a única coisa que eu noto nela é que ela tem imunidade um pouco mais baixa assim, que tem ela tem, ela tem bastante resfriado essas coisas assim né. Até agora faz uns 20 dias que a gente marcou uma consulta, falei que eu queria levar ela com a doutora Kenie, que foi a doutora que atendia ela na UTI aí né. Aí eu falei quero levar para ver o que que a doutora fala, se é assim mesmo, daí a doutora até deu risada assim, falou: “mãe ela não tem nada ela, ela é, tal, sabe?! Tá ótima...” aí ela me pediu quantas vezes ela tinha ficado internado por exemplo de pneumonias, essas coisas assim né, eu falei “não nunca”, tipo ela nunca teve nada assim. Eu que, qualquer coisinha que tem eu já me desespero né, eu acho que pelo que a gente passou, assim. Mas não, a única coisa, assim a única coisa que eu noto é que ela tem tipo, que ela pega bastante resfriado, bastante gripe, mas a doutora me falou que é normal assim, que não é nada de mal, que é da fase, dessa idade mesmo.

E: E como você avalia o atendimento da UTI Neonatal? Quais orientações você daria aos pais cujos filhos necessitam de internamento nesta unidade?

R11: O atendimento de todos é maravilhoso não existe palavras para expressar tamanha gratidão que temos por todos. Sempre fomos muito bem atendidos e a Rafaela sempre teve o melhor atendimento possível. Se pudesse falaria aos pais ficarem calmos na medida do possível pois seus filhos estão em ótima mãos.

R12

E: Qual a idade delE?

R12: 6 anos

E: Qual a altura e o peso que ele tem hoje?

R12: 17kg

E: Ela tem alguma doença ou faz uso continuado de alguma medicação?

R12: Não tem doença. Não faz uso continuo de medicamento

E: Como tem sido o acompanhamento dela? Faz no postinho de saúde? Como tem sido?

R12: Foi feito até 1 ano e meio e daí foi liberado.

Feito acompanhamento no CRE. Fez acompanhamento com Neuro.

E: Vacinas todas certinhas então, em dia?

R12: Sim, em dia

E: Qual a sua profissão?

R12: Professora

E: A renda mensal é mais ou menos de quantos salários?

R12: 1 salário

E: Após a alta da UTI, como foi o crescimento e desenvolvimento dela?

R12: Eu no começo assim eu estranhei bastante, pelo fato dele ser miudinho né, bem mais magrinho que as meninas, mas no mais falar, bom, pergunta pra sua mae pra você ver a quantia que fala, Meu Deus, demais, demais. Mas no mais assim, normal, normal, acho até por isso que liberaram ele antes né, por causa que, ele tava normal né, o médico até falou. Claro que ele é um pouco mais baixo que as outras crianças da mesma idade que ele, mas que nem o médico falou que as vezes poderia ser genética porque eu sou baixa né, o pai dele é alto mas eu sou baixa, mas no mais assim até caminhar ele caminhou na época certa, que o médico explicava que tudo ia ser bem mais tarde né pra ele falar, pra ele caminhar, gatinhar, mas a gente ia lá e o médico tinha aquela surpresa né dele falar "nossa, ele é o único né, de todos da mesma época né" ele era o única que tava se saindo normal. Não teve atraso em nada

No começo ele tomava bastante remédio que passavam eu não lembro o nome, eu tenho que olhar pra ver né, mas ele tomava bastante remédio, porque até tem pessoas que falam que as vez pode ter sido esses remédio que os dentinho dele, só os dente dele que aprodreceu muito fácil, mesmo escovando, cuidando, ele tem caries no dente, até ta

marcado pra semana que vem ta indo abiturrar, porque não é fácil pra ele ir no dentista, esse aí já não é fácil

Porque eu ouvi falar que tem crianças que as vezes fica né, diz que até na fala demora bem mais pra falar, demora bem mais pra andar, né, com o Samuel foi que nem o médico disse “ele tá reagindo como se ele não fosse prematuro, ele ta tendo o desenvolvimento de uma criança que não é prematuro” daí eu ficava pensando mas será que não ta mentindo né, eu pensava, acho que não, mas ele pra estar doente é bem difícil, tem a imunidade dele bem boa.

E: Quais interferências foram observadas no desenvolvimento do seu filho devido a utilização da UTI Neonatal?

R12: Eu acho que não, porque tipo assim a gente não entende muito né, mas eu acredito que não. Porque até falaram que a imunidade dele ia ser mais baixa, que ele ia ter problema de pulmão, e não tem porque dai eu tenho a outra que é um ano e três ou quatro meses de diferença dele e é bem difícil ficar doente, ficar engripado, bem difícil mesmo. É que falaram que a criança prematura tem a imunidade mais baixa e ele como precisou ficar ali pra ele aprender a respirar e tudo né, até eu tinha medo disso né, nossa teve noites de passar em claro pensando né, se ele ia dormir direito ou não, se ia conseguir respirar e não foi tudo normal.

Porque eu não vejo mudança assim que a gente possa falar “ah ele é diferente das outras” que nem eu tenho quatro né, três meninas e ele e eu não vejo, a única diferença que tem tipo assim é que fala demais mesmo né, fala demais mas anda, brinca, corre normal, presta atenção na hora de estar explicando, ensinando ele consegue prestar atenção ali, enxerga bem também, eu acredito que não tenha nada né, não sei eu acho que deve, eu acho que só se passar por um especialista mesmo pra, né, ta avaliando pra ver mesmo. Eu como mae assim, eu acredito que não tenha nada.

E: E como você avalia o atendimento da UTI Neonatal? Quais orientações você daria aos pais cujos filhos necessitam de internamento nesta unidade?

R12: Nossa muito bom, eu só não gostava quando tava aí era do quarto mesmo, do quarto onde a gente ficava com as outras mães lá. Mas na UTI nossa, é um cuidado muito grande, é luva, álcool, mascara mais aquele é o jaleco que fala? O avental, é um cuidado muito grande que elas tem, elas ficam em cima, pelo menos a época que eu fui aí elas ficavam bem em cima cuidando, até na hora de tirar o leite né, pra guardar pra criança, ali é cuidado bem grande.

Eu super confiei, no começo eu tinha medo né, tinha medo e daí eu tinha outra bebezinha em casa né, que no começo eu ia ficava durante o dia e a noite que eu tinha que posar ai eu vinha pra casa eu não posava aí, daí só depois quando ele foi pro quarto daí eu fiquei dia e noite, porque daí no começo né, eu vi que elas cuidavam bem que daí elas falavam "ó mae, realmente não precisa você ficar aqui por causa que ele não tava mamanda ainda no peito né" daí ela falou "Não se você quer ir pra casa" porque eu expliquei que eu tinha a outra em casa daí ela falou "não você pode ir" então eu ia e ficava o dia todo aí e a noite eu vinha pra casa, porque eu confiei muito nelas, nossa era, e quando a gente ligava a noite, passava o recado, a gente tinha o horário certinho pra falar com a medica a noite né, então eu não lembro se era as 6 ou as 7 hora que era o horário que elas passavam né, a avaliação dele durante o dia né, que mesmo quando tava lá a gente falava com a medica mas era só esse horário que elas passavam como ele tinha passado né, como tinha sido ele durante o dia ali.

Eu pelo menos eu gostava, nossa, por isso que eu vinha pra casa porque eu confiei muito ali nelas, né, porque eu vi que elas atendiam muito bem. Tinha pessoas que não gostavam, achavam que não né, mas eu não sei, as que ficavam com o Samuel eu eu me identifiquei muito com elas, eu via que cuidavam bem dele né, e ah tinha mae que ficava ali, visitavam um pouco e ficavam mais no quarto la embaixo, eu não, eu enquanto elas não falavam "ó tu pode ir" eu ficava lá mesmo, ficava la o tempo todo, que me deixavam ficar do lado dele eu ficava la, por isso que eu digo que eu gostei né, tinha mae que só ia lá, via um pouquinho e saiam, eu ficava lá.

R13

E: Qual a idade dele?

R13: Hoje ele tem 7 anos e 11 meses

E: Qual a altura e o peso que ele tem hoje?

R13: 1,17m e 23,400g

E: Ela tem alguma doença ou faz uso continuado de alguma medicação?

R13: Ele tem displasia broncopulmonar. Hje ele usa o seretide (salmeterol) uso continuo. Usou clenil por muito tempo

E: Como tem sido o acompanhamento puericultura? Faz no postinho de saúde? Como tem sido?

R13: Foi, por quase dois anos ambulatorial. Pediatra, odontologista, gastrologista, nefrologista, cirurgião urologista, pneumologista, infectologista, oftalmologista, endocrinologista

Davi nasceu com má formação.

Puericultura foi feito também

E: Vacinas todas certinhas então, em dia?

R13: Tudo ok, usou o CRIE para vacinas especiais

E: Qual a sua profissão?

R13: Professora

E: A renda mensal é mais ou menos de quantos salários?

R13: 5 salários minimos

E: Após a alta da UTI, como foi o crescimento e desenvolvimento dela?

R13: Meu Deus, o Davi é uma, uma caixinha de surpresas, ele passou alguns períodos ali bem difícieis, na questão de ganho de peso, na questão pulmonar ele teve muitas complicações, ele teve oito pneumonias num ano ali, é então a questão do ganho de peso e do desenvolvimento motor foi, é a gente sofreu um pouco. Fez fisioterapia também, por bastante tempo, é mas fez acompanhamento com mais um que eu tinha esquecido, com ortopedista. Até o ortopedista falou que provavelmente ou ele não iria andar ou ele iria demorar muito para caminhar, mas graças a Deus fez fisioterapeuta, ele andou com um ano e sete meses, pelo tamanho dele é foi um, um espanto bem grande, uma conquista muito grande porque ele era muito pequeno... de 27 e 3 (semanas)... tinha 590 gramas eu acho que ele é um dos menores do regional, até o ultimo encontro ali que a gente fez pra,

do regional ele ainda era o menor, mas acho que agora tem menores que ele ainda. Sim, 32cm e 590g. Perdeu peso e foi pra 540g ainda...

A fala, ele falou com menos de 1 ano, ele é, assim o desenvolvimento neurológico dele apesar das hemorragias e dos problemas intercorrentes que ele teve na UTI nossa foi muito bom, ele falou muito rápido, ele é, sempre foi uma criança que soube se expressar muito bem, ali o que a gente teve mais dificuldade e ainda tem um pouquinho de luta que acompanha, hoje ele acompanha com a PO e a psicopedagoga é aa questão motora fina, que ele ainda tem um pouquinho de dificuldade em alguns movimentos, é pra escrever, ele tem, ele não tem a letra bonita como a maioria das crianças, ele tem dificuldade espacial, é e a gente investida um TDAH também, mas assim, a gente sempre levou em todos os profissionais que foi indicado, então ele teve acompanhamento e perto do que ele era perto das outras crianças o desenvolvimento dele foi muito bom, ele não, depois que ele começou a caminhar ele não caminhou mais ele só correu. Ele era um foguetinho, e sim, muito ativo. Tinha dificuldade no ganho de peso, apesar de comer sempre muito bem, é porque ele era muito ativo, ele não parava nem pra dormir.

...Na verdade eu acho que era mais medo do que, porque como ele era muito pequeno a gente tinha muito medo dele não sobreviver. E ele teve hemorragia cerebral, hemorragia pulmonar, parada cardio, falência de órgãos, foi uma trajetória bem difícil. É até hoje a gente acompanha com o neuro também pra ver essa questão do desenvolvimento, o neuro fala que o cérebro dele é lindo, pra quem teve hemorragia cerebral, sim, nossa ele é uma criança incrível, muito ativo, muito falante, assim tem um desenvolvimento acima da faixa etária dele assim até, pelo que ele passou.

A gente sempre fala que ele foi tao apressado que, e daí ele era gemelar, o mano faleceu intrauterino daí a gente até, brinca que ele tem dois por dentro, ele e o mano...

...Sim, foi cesárea, aham, foi por que o mano faleceu a gente descobriu na sexta-feira e fez a minha cesárea na terça feira, mas nesse meio tempo a gente também descobriu que o Davi tinha inserção velamentosa então isso é uma das causas do, é, dele não tem o tamanho ideal pra faixa ali da semana né, o tempo gestacional.

E: Quais interferências foram observadas no desenvolvimento do seu filho devido a utilização da UTI Neonatal?

R13: Não, a princípio a questão pulmonar ali que ele tem um pulmão mais frágil mas é pela, pelo tempo né que ele nasceu, por tanto tempo no oxigênio, ele ficou 98 dias no oxigênio, então, é o próprio pneumo fala, comentava que o próprio oxigênio que ajudou ele a sobreviver foi o que também prejudicou o pulmão né, então, mas fora isso acho que,

que a questão também talvez emocional, ele é uma criança que tem que ter sempre alguém meio por perto, ele tem medo da separação, da perda, mas que a gente associa ao tempo que ficou sozinho na NEO. Foi demorado pra pegar o primeiro colo, o primeiro mama no peito, é então tudo com ele demorou... Ele ficou 107 dias na UTI.
O pneumo nos deu alta agora no inicio do ano, falou que as intercorrências que acontecerem de agora pra frente é porque ele, é por ser criança, é por mudanças climáticas, claro que a gente tem que ter um cuidado a mais porque o pulmão dele não é 100% igual o das outras crianças, mas que é vida normal pra ele bem tranquilo, ele pode brincar, correr fazer todos os tipos de atividades físicas. Nossa, ele é nosso milagre.
Não e se for ver ele não tem nada, ele é perfeito.
E: E como você avalia o atendimento da UTI Neonatal? Quais orientações você daria aos pais cujos filhos necessitam de internamento nesta unidade?
R13: Meu Deus, eu chego a me emocionar sabe, porque é nota mil assim, a nota máxima que existir o máximo dos máximos. Porque além de acolher ele, de cuidar dele eles sempre tiveram o cuidado conosco também, com a família. Assim, em todos os sentidos, no ensinar a cuidar, no é, no mamazinho, no carinho de dar o mamazinho, carinho do toque, assim, em muitos momentos eu não pude, eu não podia estar ali, que ainda não era aberto livre, não sei como que tá hoje, mas a gente tinha os períodos de, de visita, é ali mais pro final quando ele tava um pouquinho maior já, começou a ser liberado a mae ficar, permitido a ficar mais tempo, então eu pude ficar ali na UTI mais tempo com ele mas quando eu não tava é as meninas cuidavam como se fossem delas, e quando ele aprendeu o que que era colo ele gostou do colo, então ele chorava que queria ficar no colo, e elas davam esse colo pra ele, davam todo o amor e atenção, carinho como se fosse, é, quando eu não podia estar presente. Nossa, como um todo, todos elas, maravilhosas.
Ai, que tenham fé e não desistam porque o que é da gente só a gente que passa e com certeza todas essas crianças que passam por isso elas têm um papel que vao cumprir na sociedade um papel muito importante. Elas já vêm com uma missão diferente, elas vem para nos ensinar algo novo, é, não desistir, tenham fé, escutem as médicas, escutem os enfermeiros, que eles tao passando além da experiencia deles, eles tao passando o que que o filho da gente precisa, é, oração, correntes de oração, muito carinho, porque as crianças ali de dentro precisam de carinho, precisam da atenção, do afeto, da família acho que isso faz toda a diferença. Tenham fé e paciência, porque os dias parecem longos, são muito longos dentro da UTI, mas eles chegam ao fim também, e a coisa mais maravilhosa do mundo é voltar com as crianças pra casa, é uma emoção sem tamanho. Tem dias bons,

tem dias ruins, tem dias mais ruins, mas tem os dias que a cada dez gramas que ganham a gente sai saltitando, é uma comemoração a gente ali na UTI acaba se sentindo parte da família porque a gente passa mais tempo ali do que em casa, a gente tem mais contato, então, meu Deus é, não tenho nem palavras pra equipe. Davi começou com 1ml então eu lembro, eu lembro como eu comemorava cada, cada gramazinha, eu chegava e já olhava pro quadro pra ver quantos, quanto que ele tava mamando e quantos gramas tinha ganhado.

Teve muita intercorrência, por isso que eu falo, tenham fé porque a fé move montanhas a gente não é nada sem a fé, sem acreditar que vai melhorar, que vai dar tudo certo, o pensamento positivo também, se apeguem nas pessoas que tem confiança, a gente sempre também precisa da, tá tao na moda falar a rede de apoio em casa né, mas é muito importante, uma mae saudável um pai saudável e forte é, consegue cuidar do filho, é com mais força, então eu falo por mim, se eu não to bem, como é que eu vou cuidar do meu filho, então os pais precisam se cuidar também, é muito importante para, pra tá bem pra cuidar dos seus filhos.
